# Enhancement of CEP215 dynamics for spindle pole assembly during mitosis

**DOI:** 10.1242/jcs.263542

**Published:** 2025-05-21

**Authors:** Hyunjeong Yoo, Taehyun Kim, Sungjin Ryu, Donghee Ko, Jeesoo Kim, Hee-Jung Choi, Yongdae Shin, Kunsoo Rhee

**Affiliations:** ^1^ Department of Biological Sciences, Seoul National University, Seoul 08826, Korea; ^2^ Department of Mechanical Engineering, Seoul National University, Seoul 08826, Korea

**Keywords:** CEP215, CDK5RAP2, Centrosome, Pericentriolar material, Mitotic spindle, Spindle pole, Protein dynamics

## Abstract

The microtubule-organizing activity of centrosomes fluctuates during the cell cycle, reaching the highest levels at M phase. CEP215 (also known as CDK5RAP2) is a key pericentriolar material (PCM) protein for microtubule organization of the human centrosome. Here, we provide evidence that CEP215 exhibits a dynamically suppressed, solid-like state in interphase centrosomes, and becomes a more dynamic state in mitotic centrosomes. Specific interaction with PCNT, another centrosome protein, is crucial for diffusible molecular dynamicity of the CEP215 protein. We also found that the cluster formation activity of CEP215 is impaired in a light-inducible system when its coiled-coil domains (CCDs) are truncated. Defects in spindle pole assembly and spindle formation were accompanied in the cells whose CEP215 is replaced with the CCD-truncated mutants. Our results support the notion that the diffusible mobility of CEP215 is enhanced by both homotypic and heterotypic interactions among CCDs, especially at mitotic spindle poles. This work highlights that biophysical properties of the PCM proteins at the centrosomes fluctuate during the cell cycle.

## Teaser

**Summary:** The molecular dynamics of CEP215 fluctuates at the centrosomes and is highest at M phase. Multimeric interactions among the coiled-coil domains are likely important for enhancement of diffusible mobility of CEP215.

## INTRODUCTION

Recent studies have demonstrated that biomolecules can often undergo phase separation processes, which serve as a biogenesis mechanism for several non-membranous organelles (Alberti, 2017; Boeynaems et al., 2018). Through a network of intermolecular interactions, a set of proteins and nucleic acids can phase-separate from other molecules to form coexisting phases, each of which is distinct in composition (Shin, 2022). Resulting dense phases, i.e. condensates, often exhibit liquid-like material properties with dynamic rearrangements of constituent molecules, although examples of solid-like condensates are not uncommon (Shin et al., 2017). Condensate assembly and disassembly can be dynamically regulated during cell cycle and signaling through various mechanisms, such as post-translational modification, the availability of RNA and changes in physicochemical conditions of cells (Alberti and Hyman, 2021). Aberrant types of phase separation have been associated with diverse diseases, including cancers and neurodegenerative diseases (Alberti and Dormann, 2019; Mensah et al., 2023).

Phase separation mechanisms have been implicated in assembly of centrosomes, which are non-membranous organelles (Woodruff, 2021). A centrosome consists of a pair of centrioles surrounded by a protein-rich matrix known as pericentriolar material (PCM). The microtubule-organizing activity of centrosomes changes during the cell cycle, such that the size of centrosomes robustly increases when a cell enters M phase and reduces to a minimum once the cell exits mitosis. Given that the PCM is the place that organizes most microtubules at the centrosome, its levels dynamically fluctuate during the cell cycle, reaching the highest at M phase, and disintegrating from the centrosomes after mitosis. It is suspected that phase separation is involved in recruitment and retention of the PCM proteins at M phase (Woodruff, 2021). However, a large amount of experimental evidence also supports a traditional view that centrosomes are assembled upon a more solid, stable scaffold through high-affinity interactions (Raff, 2019).

SPD-5, a PCM protein in *C. elegans*, stands out as a pioneer centrosome protein exhibiting phase separation capabilities. SPD-5 phase separation is facilitated by macromolecular crowding and multivalent homotypic interactions *in vitro* (Woodruff et al., 2017). The phase-separated SPD-5 is able to organize microtubules *in vitro* along with a few additional proteins (Woodruff et al., 2017). Among the mammalian centrosome proteins, the CEP63–CEP152 complex (Ahn et al., 2020), CEP57 (Yeh et al., 2024) and a fragment of PCNT (Jiang et al., 2021), have been identified as undergoing phase separation. However, it remains largely elusive whether phase separation behaviors of individual centrosome components contribute to centrosome assembly and functions in living cells.

CEP215 (also known as CDK5RAP2), a human homolog of SPD-5, is a major PCM protein for microtubule organization at the centrosome. The amount of centrosome CEP215 is regulated during the cell cycle, being the highest at M phase (Haren et al., 2009; Lee and Rhee, 2010). In this study, we probe the molecular dynamicity of CEP215 during the cell cycle stages. We found that a combination of homotypic interactions between CEP215 and heterotypic interactions with PCNT play a crucial role in organizing proper spindle pole assembly during mitosis, by facilitating molecular dynamics and the accumulation of PCM.

## RESULTS

### Diffusive dynamics of CEP215 at the centrosomes

**Fig. 1.**
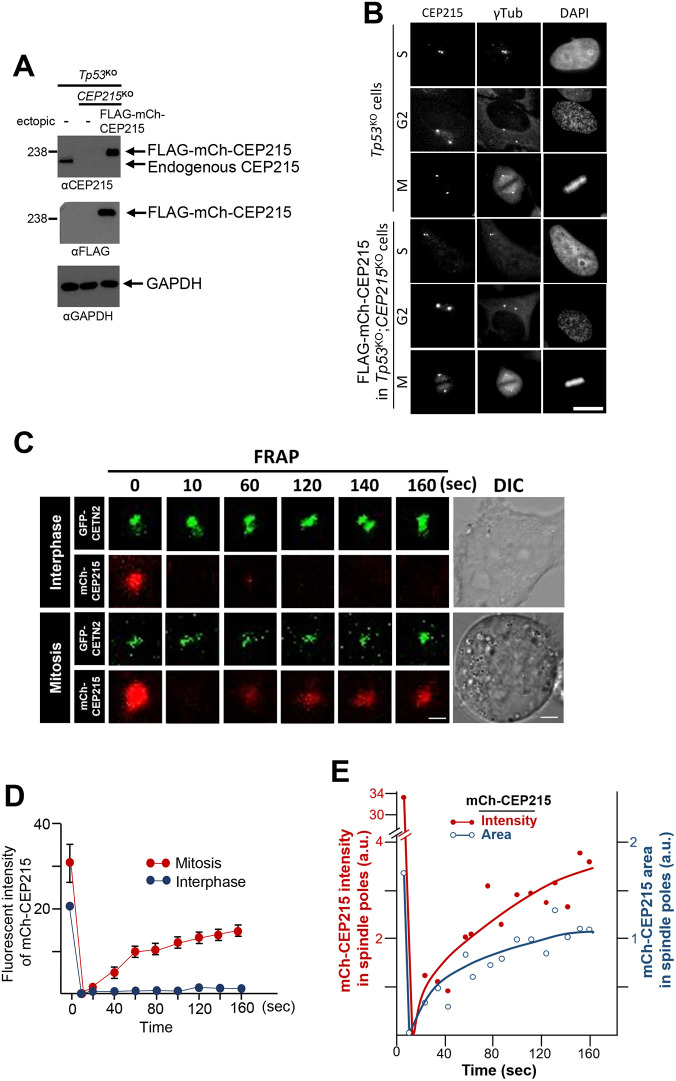
**FRAP analyses of FLAG–mCh–CEP215 at the interphase and mitotic centrosomes.** (A) FLAG–mCh–CEP215 protein was stably expressed in *p53*^KO^;*CEP215*^KO^ HeLa cells, and subjected to immunoblot analyses with anti-CEP215, FLAG, and GAPDH antibodies. Blot representative of three experimental repeats. (B) FLAG–mCh–CEP215-rescued HeLa cells were co-immunostained with anti-CEP215 and γ-tubulin antibodies. DNA was stained with DAPI. Images representative of three experimental repeats. Scale bar: 10 μm. (C) The FLAG–mCh–CEP215 proteins in *CEP215*-deleted HeLa cells were subjected to FRAP analyses at interphase and mitotic phase centrosomes. GFP–centrin-2 marks the centrosomes. The analyzed cells were also photographed with differential interference contrast (DIC) images. Scale bars: 1 μm (FRAP); 10 μm (DIC). (D) FRAP signals of the FLAG–mCh–CEP215 at the centrosomes were measured for up to 160 s after the bleaching. At least 10 cells per group were analyzed in three independent experiments. Values are mean±s.e.m. and are arbitrary units. (E) Intensities and areas of the FLAG–mCh–CEP215 signals were measured during the FRAP analysis at mitotic spindle poles in C. The solid lines represent regression curves generated using the Prizm software. a.u., arbitrary units.

We first sought to probe the mobility of CEP215 within the centrosome in living cells. To this end, we induced the expression of FLAG–mCh–CEP215 with doxycycline in *CEP215*-knockout (KO) HeLa cells. The expression levels of ectopic FLAG–mCh–CEP215 were ∼1.7 times higher than endogenous CEP215 in control cells (Fig. 1A). Similar to endogenous CEP215, FLAG–mCh–CEP215 was localized to the centrosomes. The accumulation levels of FLAG–mCh–CEP215 change during the cell cycle, such that it was at a minimum at S phase, started to accumulate to a maximum at G2 phase, and somewhat reduced at M phase, as previously reported by us (Lee and Rhee, 2011; Fig. 1B). We then examined the molecular mobility of CEP215 in the centrosome using a fluorescence recovery after photobleaching (FRAP) assay. We found that CEP215 exhibited drastically different degrees of fluorescence recovery, depending on the cell cycle stage (Fig. 1C). FLAG–mCh–CEP215 in mitotic cells showed fast rearrangement, whereas those in the interphase cells exhibited minimal recovery (Fig. 1D). This suggests that the molecular dynamics of CEP215 in the centrosomes are highly variable, such that they are at the dynamically arrested state in interphase and becomes highly mobile during mitosis. The fluorescent signaling area of FLAG–mCh–CEP215 swiftly recovered to reach to a half of original area in 120 s, suggesting that mCh–CEP215 might be exchanged, irrespective of the relative positions within the spindle poles (Fig. 1E).

### Light-induced clustering of ectopic mCh–CEP215–CRY2

**Fig. 2.**
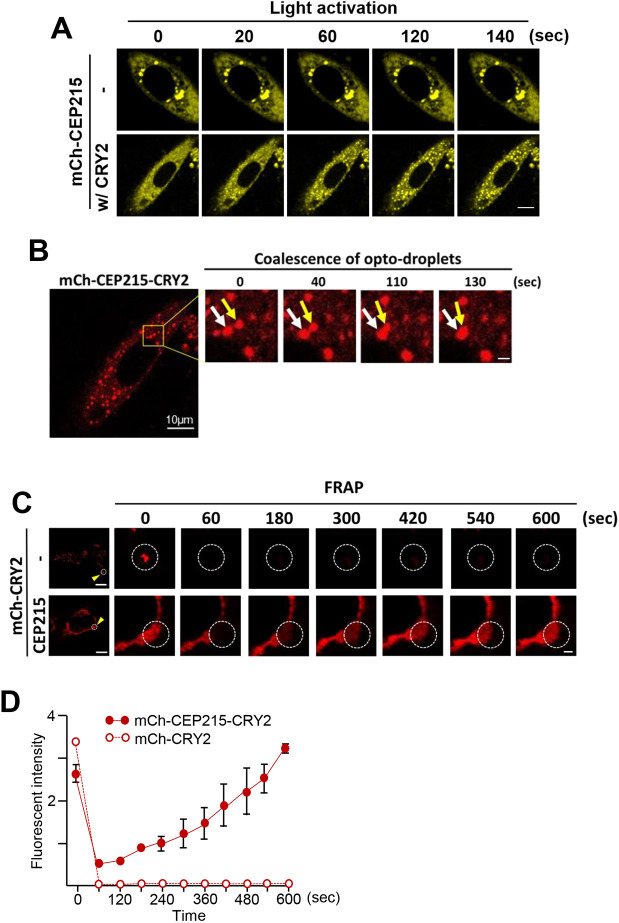
**Cluster formation of mCh–CEP215–CRY2 under light activation.** (A) mCh–CEP215 with (w/) and without (-) the CRY2 domain were transiently expressed in HeLa cells and light activated for up to 140 s (Movie 1). (B) Coalescence of the mCh–CEP215–CRY2 clusters (arrows) after the light activation. (C) FRAP analysis was performed at the mCh–CRY2 and mCh–CEP215–CRY2 clusters. Circles indicate bleached regions. Scale bars: 10 μm (A, overview images in B,C); 1 μm (magnifications in B,C). (D) The FRAP signals of mCh–CRY2 (open circle) and mCh–CEP215–CRY2 (filled circle) were measured for up to 600 s. At least 30 cells per experimental group were counted in three independent experiments. Values are mean±s.e.m. and are arbitrary units.

We employed the opto-droplet system to reconstitute the complexity of biomolecular clusters in live cells by induction of protein multimerization with external light input (Shin et al., 2017; Wei et al., 2020; Lee et al., 2024a). Our opto-droplet construct mCh–CEP215–CRY2 comprises CEP215 fused to mCherry (mCh) for imaging and the CRY2 domain for multimerization by light activation. The construct was transiently transfected into HeLa cells and live-cell imaging was undertaken by confocal microscopy. mCh–CEP215–CRY2 signals were visible in the cytoplasm, in addition to the centrosomes (Fig. 2A). Live-imaging revealed that mCh–CEP215–CRY2 instantly formed clusters in the cytoplasm upon light activation (Fig. 2A; Movie 1). By contrast, mCh–CEP215 had already formed aggregates, which with diverse shapes and sizes, before the light activation (Fig. 2A). The light-induced mCh–CEP215–CRY2 clusters showed characteristics of liquid-like condensates. They often grew in size through fusion and showed shape relaxation behaviors after fusion (Fig. 2B). FRAP measurements also revealed highly mobile molecular dynamics within light-induced CEP215 condensates (Fig. 2C,D).

We then examined the concentration dependence of light-induced clustering of mCh–CEP215–CRY2. To facilitate data acquisition, we adopted immunostaining analyses of FLAG–mCh–CEP215–CRY2 in fixed cells (Fig. S1A). When compared across the cells expressing different levels of mCh–CEP215–CRY2, a strong correlation between expression levels and the degree of clustering was observed (Fig. S1B). The fraction of cluster-positive cells increased as the expression level increased. Cells expressing low levels of CEP215 showed dispersed signals, whereas high expression of CEP215 led to the formation of cytoplasmic aggregates irrespective of the presence the CRY2 domain (Fig. S1B,C). However, in the cells expressing moderate levels, FLAG–CEP215–CRY2 cluster formation was induced upon light activation, while the majority of FLAG–CEP215 remained diffusive in the cytoplasm (Fig. S1C). Taken together, the opto-droplet assay demonstrates that CEP215 undergoes light-induced clustering when linked to the CRY2 domain.

### PCNT interaction is important for diffusible dynamics of CEP215

**Fig. 3.**
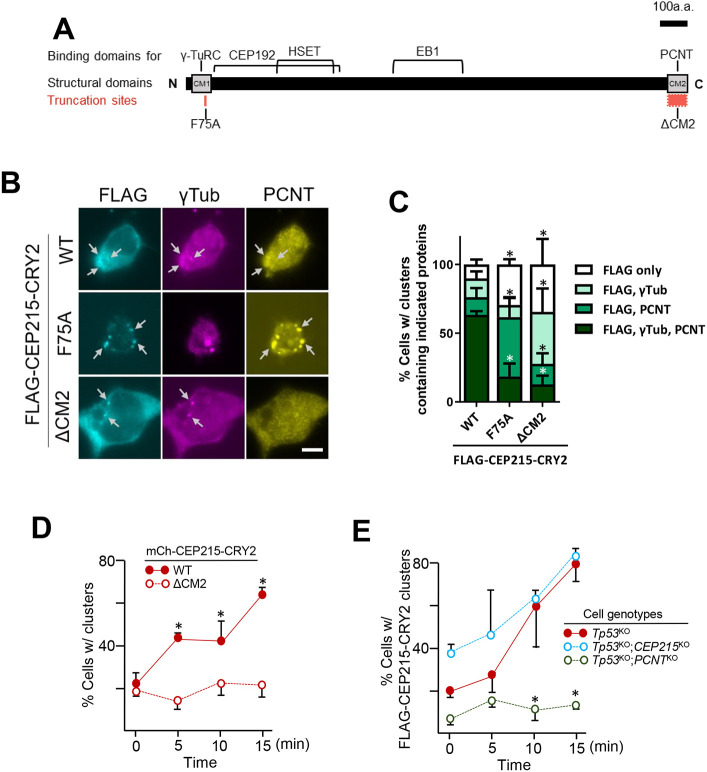
**Involvement of PCNT in cytoplasmic cluster formation of FLAG–CEP215–CRY2.** (A) Schematic diagram illustrating the CEP215 protein with functional sites (CM1 and CM2), interaction domains for PCM proteins and the mutants used in this study (F75A and ΔCM2). (B) HeLa cells expressing FLAG–CEP215–CRY2 (WT, F75A and ΔCM2) were light-activated for 10 min, and co-immunostained with FLAG (cyan), γ-tubulin (magenta) and PCNT (yellow) antibodies. Cytoplasmic clusters are marked with arrows. Scale bar: 10 μm. (C) The number of cells with (w/) the cytoplasmic clusters containing γ-tubulin and PCNT signals was counted. (D) The FLAG–CEP215–CRY2 (WT and ΔCM2)-expressing cells were light-activated for up to 15 min and the number of cells with cytoplasmic clusters was counted. (E) FLAG–CEP215–CRY2 was transiently expressed in the *p53*^KO^ (red filled circle), *p53*^KO^;*CEP215*^KO^ (blue open circle) and *p53*^KO^;*PCNT*^KO^ (green open circle) cells. The cells were light-activated and the number of cells with cytoplasmic clusters of FLAG-CEP215-CRY2 was counted. Representative immunostaining images are presented in Fig. S2B. For C–E, at least 90 cells per experimental group were counted in three independent experiments. Values are mean±s.e.m. *P<0.05. Statistical significance was determined using one-way ANOVA with Tukey's post hoc compared with WT (for C,D) or *p53*^KO^ (for E).

We then examined whether light-induced clusters of CEP215 can recruit other components of centrosomes, such as γ-tubulin and PCNT, which are known to have high-affinity interactions with CEP215 (Fig. 3A). Immunostaining experiments showed that both γ-tubulin and PCNT were colocalized at the cytoplasmic clusters of FLAG–CEP215–CRY2 (Fig. 3B), but not in the aggregates (data not shown). In fact, microtubules might be organized from the cytoplasmic clusters of FLAG–CEP215–CRY2 (Fig. S2A).

CEP215 includes two distinct functional domains, CM1 and CM2 (Fig. 3A). The CM1 domain has been shown to interact with γ-tubulin ring complex (γTuRC) for microtubule organization (Fong et al., 2008). Replacement of phenylalanine at 75th residue with alanine (F75A) perturbs the molecular association with γTuRC (Choi et al., 2010). The CM2 domain is important for centrosome localization of CEP215, and the deletion of the CM2 domain (ΔCM2) diminishes the interaction of CEP215 with other centrosome proteins, such as AKAP450 and PCNT (Kim and Rhee, 2014; Wang et al., 2010). Our analysis shows that ∼60% of cells containing FLAG–CEP215–CRY2 clusters displayed both γ-tubulin and PCNT signals, whereas fewer than 20% of cells expressing FLAG–CEP215^F75A^–CRY2 or FLAG-CEP215^ΔCM2^–CRY2 contained both signals in the clusters. (Fig. 3C). The majority of the FLAG–CEP215^F75A^–CRY2 and FLAG–CEP215^ΔCM2^–CRY2 clusters were selectively devoid of γ-tubulin and PCNT, respectively (Fig. 3C). Furthermore, the microtubule-organizing activity was also reduced from cytoplasmic clusters of FLAG–CEP215^ΔCM2^–CRY2 (Fig. S2A). These results suggest that CEP215 in cytoplasmic clusters is able to interact with PCM proteins in a sequence-specific manner.

As we examined the light-induced clustering of CEP215 variants, we noticed that in the absence of CM2 domain, mCh–CEP215–CRY2 exhibited a minimal light-dependent condensation, suggesting a critical role of the CM2 domain on CEP215 clustering in cells (Fig. 3D). This result also implies that the association of PCNT, rather than the CM2-mediated self-association per se, may play an important role in the CEP215 cluster formation. To distinguish between these possibilities, we transiently expressed FLAG–CEP215–CRY2 in the *CEP215*^KO^ and *PCNT*^KO^ cell lines. The results showed that cytoplasmic clusters of FLAG–CEP215–CRY2 were formed in *CEP215*^KO^ cells after light activation, but not in the *PCNT*^KO^ cells (Fig. 3E; Fig. S2B). These results suggest that PCNT interaction at the CM2 domain of CEP215 is crucial for its cytoplasmic cluster formation.

**Fig. 4.**
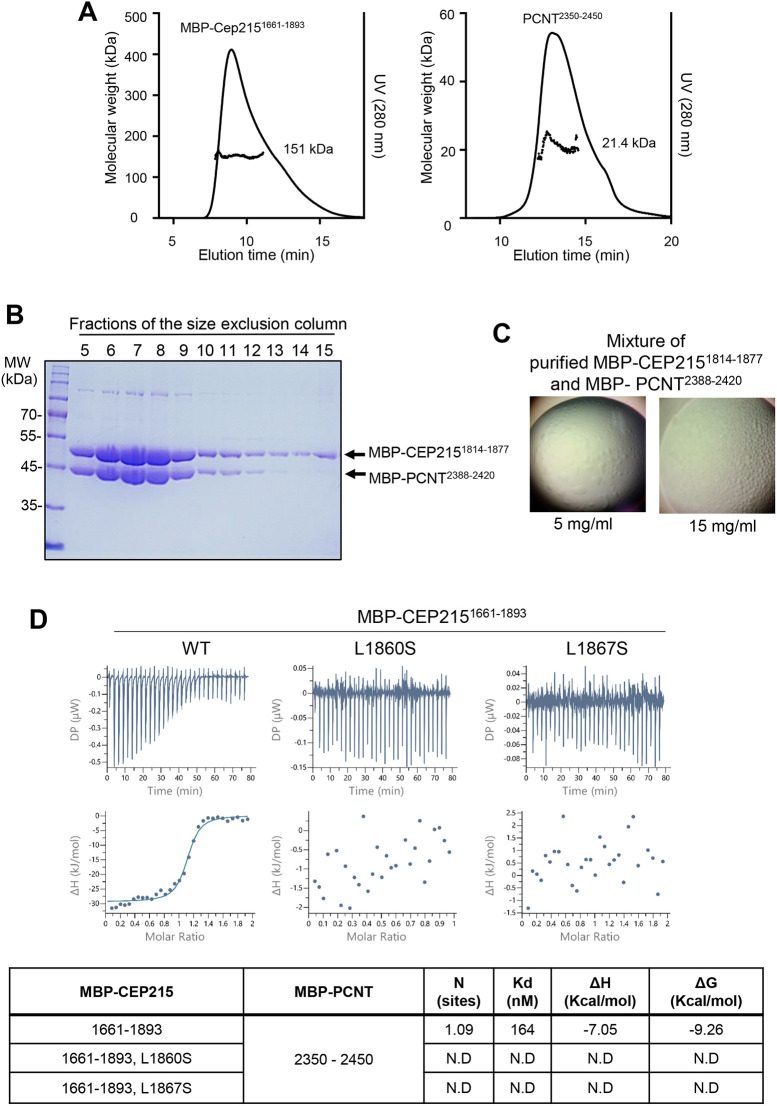
**Biochemical characterizations of the CEP215 interaction with PCNT.** (A) SEC-MALS analysis of MBP–CEP215^1661-1893^ and PCNT^2350-2450^. The calculated molecular mass of each monomeric fragment based on its amino acid sequence is 71.8 kDa for MBP–CEP215^1661-1893^ and 11.3 kDa for PCNT^2350-2450^, indicating that they exist as dimers in solution. (B) Size exclusion chromatography of the mixture of purified MBP–CEP215^1814-1877^ and MBP–PCNT^2388-2420^, followed by SDS-PAGE. Both the CEP215 and PCNT proteins were eluted in the same fractions. (C) Under crystallization screening conditions, the mixture of purified MBP–CEP215^1814-1877^ and MBP–PCNT^2388-2420^ exhibited liquid-like droplets but failed to form crystals. (D) Isothermal titration calorimetry analysis of L1860S and L1867S point mutants of MBP–CEP215^1661-1893^ with MBP–PCNT^2388-2420^. The upper panel shows raw data for the wild-type and point mutants of MBP-CEP215^1661-1893^, and the bottom panel displays the corresponding binding curves. The dissociation constants (*K*_d_) and thermodynamic parameters for each interaction are summarized in the table. N.D., not determined. Results in this panel representative of two experimental repeats.

We analyzed physical interactions of CEP215 and PCNT *in vitro*. Size-exclusion chromatography with multi-angle light scattering (SEC-MALS) analysis revealed that MBP–CEP215^1661-1893^ and PCNT^2350-2450^ form homodimers in solution (Fig. 4A). When MBP–CEP215^1814-1877^ and MBP–PCNT^2388-2420^ were mixed in solution and subjected to the SEC analysis, both the proteins were eluted at the same fractions, suggesting that they instantly form a heterogenous complex *in vitro* (Fig. 4B). However, the mixture did not form crystals even at high concentrations, suggesting that the complex might adopt a flexible configuration (Fig. 4C). This flexibility might also be important for the dynamic behavior of the complex in the cellular context. Isothermal titration calorimetry (ITC) analyses indicated that CEP215 and PCNT fragments interact in an equal ratio with a dissociation constant of 164 nM (Fig. 4D).

**Fig. 5.**
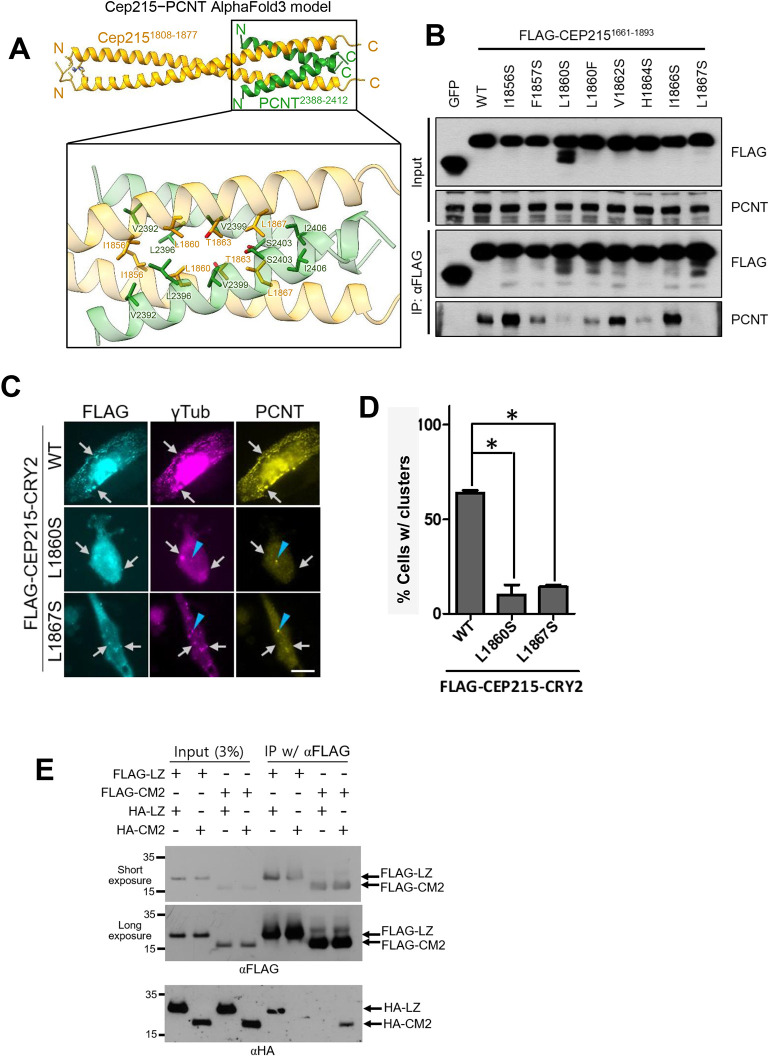
**Characterization of the homotypic and heterotypic interactions CEP215.** (A) The tetrameric structure of the CEP215–PCNT complex, predicted by AlphaFold3. CEP215^1808-1877^ dimer (yellow) and PCNT^2388-2412^ dimer (green) interact through their respective regions. Enlarged view highlights key residues involved in the interaction. (B) The FLAG–CEP215^1661-1893^ proteins with indicated point mutations were expressed in 293T cells and immunoprecipitated with the FLAG antibody followed by immunoblot analyses with anti-FLAG and PCNT antibodies. Input, 2%. Blot representative of three experimental repeats. (C) The FLAG–CEP215–CRY2 point mutant-expressing cells were light-activated, and co-immunostained with anti-FLAG (cyan), γ-tubulin (magenta) and PCNT (yellow) antibodies. Centrosomes and FLAG–CEP215 clusters are marked with arrowheads and arrows, respectively. Scale bar: 10 μm. (D) The number of cells with (w/) cytoplasmic clusters was counted. At least 90 cells per experimental group were counted in three independent experiments. Values are mean±s.e.m. *P<0.05 (one-way ANOVA with Tukey's post hoc test). (E) The FLAG- and HA-tagged LZ (470–560) and CM2 (1661–1863) domains of CEP215 were transiently expressed in 293T cells, immunoprecipitated with anti-FLAG antibody, and immunoblotted with the FLAG and HA antibodies. Blot representative of two experimental repeats.

A predicted complex using AlphaFold3 reveals that the two proteins engage in hydrophobic interactions at the core of the coiled-coil structure in a leucine zipper configuration (Fig. 5A; Fig. S3). Specifically, L1860 and L1867 of CEP215 interact directly with L2396, V2399 and I2406 of PCNT (Fig. 5A). We performed co-immunoprecipitation experiments to determine specific interactions of FLAG–CEP215^1661-1893^ with endogenous PCNT. The immunoblot analyses revealed that endogenous PCNT was coimmunoprecipitated with FLAG–CEP215^1661-1893^, but not with the L1860S and L1867S point mutants (Fig. 5B). Co-immunostaining analyses also revealed that PCNT is placed at the centrosomes but can hardly be detected at the cytoplasmic clusters of FLAG–CEP215^L1860S^–CRY2 and FLAG–CEP215^L1867S^–CRY2 (Fig. 5C). Furthermore, neither FLAG–CEP215^L1860S^–CRY2 nor FLAG–CEP215^L1867S^–CRY2 generated cytoplasmic clusters efficiently (Fig. 5D). These results strongly suggest that PCNT interaction is essential for formation of CEP215 clusters.

It has been previously reported that centrosomin (Cnn), a *Drosophila* homolog of CEP215, forms homo-multimers (Feng et al., 2017). Especially, a homotypic interaction between the conserved leucine zipper (LZ) and CM2 domains of Cnn is crucial for PCM scaffolds in *Drosophila* mitotic centrosomes (Feng et al., 2017). Therefore, we performed co-immunoprecipitation assays with CEP215^611-670^ and CEP215^1661-1893^, which potentially correspond to the LZ and CM2 domains of human CEP215, respectively (Feng et al., 2017). First, we observed that both CEP215^611-670^ and CEP215^1661-1893^ formed homo-multimers, suggesting they contribute to homomultimer formation of CEP215 (Fig. 5E). However, HA–CEP215^611-670^ was not co-immunoprecipitated with FLAG–CEP215^1661-1893^, nor was HA–CEP215^1661-1893^ with FLAG–CEP215^611-670^ (Fig. 5E). These results suggest that the human CEP215 might not form the LZ–CM2 tetramer assembly that had been observed with *Drosophila* Cnn (Feng et al., 2017).

### Isolation of CEP215 mutants with impaired molecular dynamicity

**Fig. 6.**
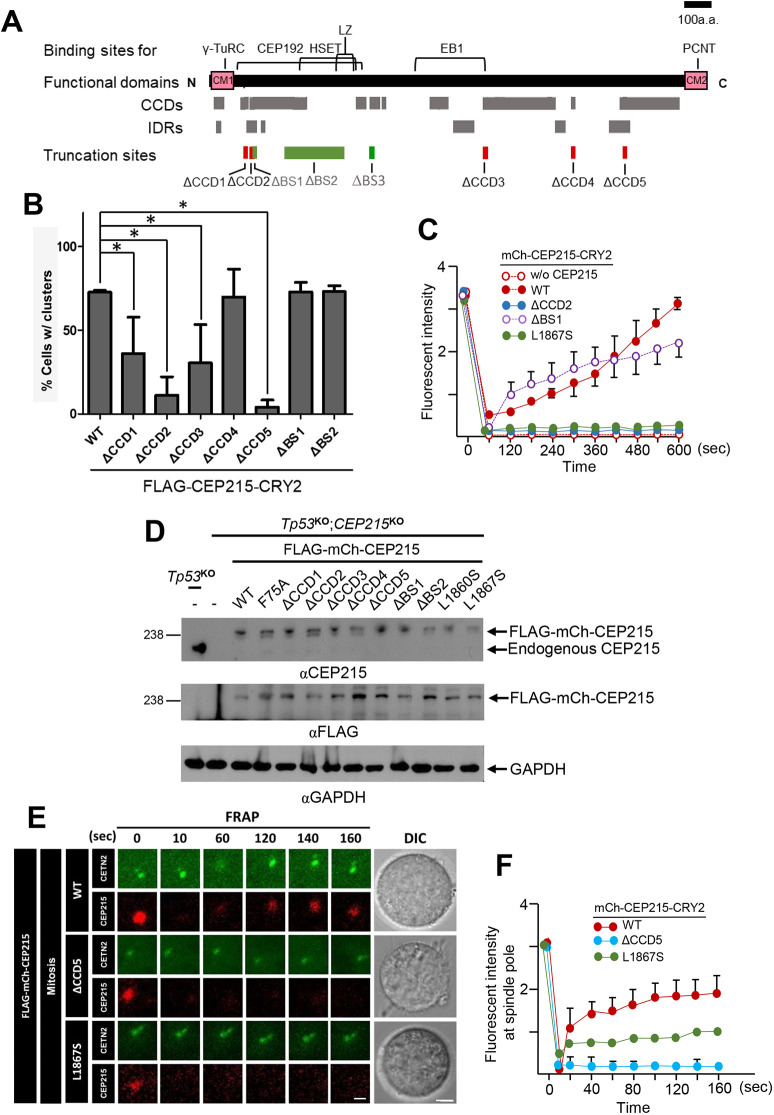
**Generation of CEP215-truncated mutants with defects in molecular dynamicity.** (A) Schematic diagram illustrating the CEP215 protein with functional sites (CM1 and CM2), protein-binding sites, coiled-coil domains (CCDs), intrinsically disordered regions (IDRs) and the truncated mutant sites used in this study. (B) The number of cells with (w/) cytoplasmic clusters of FLAG–CEP215–CRY2-truncated mutant proteins was counted. *P<0.05 (one-way ANOVA with Tukey's post hoc test). (C) FRAP signals of the mCh–CEP215–CRY2 mutant proteins at the cytoplasm were measured for up to 600 s. w/o, without. (D) Stably expressed FLAG–mCh–CEP215 mutants in *p53*^KO^;*CEP215*^KO^ HeLa cells were subjected to immunoblot analyses with anti-CEP215, FLAG and GAPDH antibodies. Blot representative of four experimental repeats. (E) FRAP analyses of the FLAG–mCh–CEP215 proteins were carried out at mitotic phase centrosomes of the rescued cells. GFP–centrin2 marks the centrosomes. The analyzed cells were photographed with differential interference contrast microscopy. Scale bars: 1 μm (FRAP); 10 μm (DIC). (F) The FRAP signals of FLAG–mCh–CEP215 (WT, ΔCCD5 and L1867S) at mitotic phase centrosomes were measured for up to 160 s. For B, C and F, at least 90 (B), 30 (C) and 10 (F) cells per experimental group were counted in three independent experiments. Values are mean±s.e.m. C and F show arbitrary units.

CEP215 includes multiple coiled-coil domains (CCDs), some of which are known to participate in protein–protein interactions (Rios et al., 2024). Indeed, CEP215 is known to interact with a number of proteins, such as γ-TuRC, CEP192, HSET, EB1 and PCNT (Chavali et al., 2016; Fong et al., 2009; Kim and Rhee, 2014; Kuriyama and Fisher, 2020) (Fig. 6A). We generated CEP215 mutants in which selected CCDs (ΔCCD1–5) and protein–binding sites (ΔBS1–3) are truncated. We found that endogenous PCNT and CEP192 were co-immunoprecipitated with the selected FLAG–CEP215 mutants, suggesting that the CCD-truncated mutants do not affect known sequence-specific interactions of CEP215 with the PCM proteins (Fig. S4). Consistent with this view, CEP192 and PCNT were not co-immunoprecipitated with ΔBS1 and L1867S mutants of FLAG–CEP215, which lack corresponding binding domains (Fig. S4).

We observed that the CCD-truncated FLAG–CEP215–CRY2 mutants did not form cytoplasmic clusters efficiently except for ΔCCD4, whereas those truncated at the CEP192- and HSET-binding sites (ΔBS1,2) efficiently formed cytoplasmic condensates (Fig. 6B; Fig. S5A). FRAP analysis revealed that fluorescence signals of the CCD-truncated mCh–CEP215–CRY2 mutants recovered poorly, whereas those of mCh–CEP215^ΔBS1^–CRY2 and mCh–CEP215^ΔBS2^–CRY2 recovered efficiently (Fig. 6C; Fig. S6). Fluorescence of the PCNT-interaction-deficient mCh–CEP215^L1860S^–CRY2 and mCh–CEP215^L1867S^–CRY2 proteins also did not recover efficiently (Fig. 6C; Fig. S6). These results suggest that the clustering ability of CEP215 relies on intact CCD structures and interaction with PCNT.

### Defects in spindle pole assembly and spindle formation in mitotic cells with the CCD-truncated CEP215 mutants

**Fig. 7.**
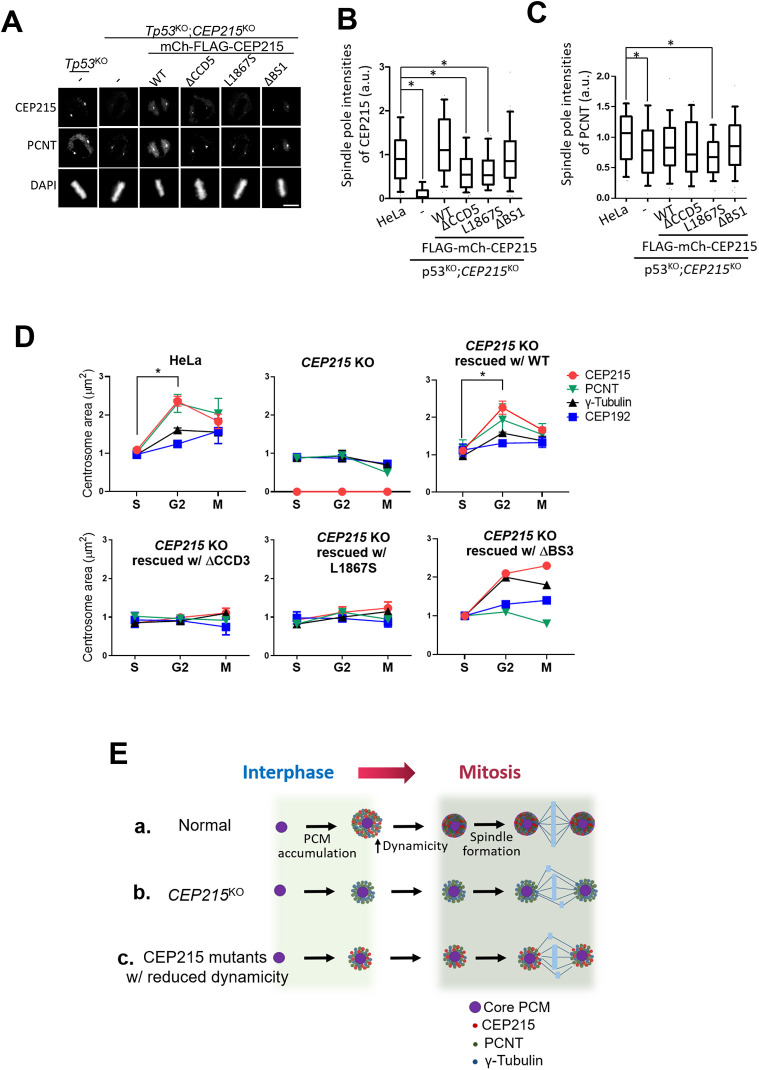
**Spindle pole recruitment of the FLAG–mCh–CEP215 mutant proteins.** (A) The FLAG–mCh–CEP215 mutant replacement HeLa cells were treated with proTAME for the metaphase arrest. The cells were co-immunostained with anti-CEP215 and PCNT antibodies. DNA was stained with DAPI. Scale bar: 10 μm. (B,C) Spindle pole intensities of FLAG–mCh–CEP215 (B) and endogenous PCNT (C) were measured and presented as box plots. (D) Cell cycle of the FLAG–mCh–CEP215 mutant replacement cells were synchronized with a double-thymidine block-and-release method and co-immunostained with anti-CEP215, PCNT, γ-tubulin and CEP192 antibodies. The area of the specific PCM proteins were measured. For B–D, at least 50 cells per experimental group were counted in three independent experiments. In B and C, box represents the 25–75th percentiles, and the median is indicated. The whiskers show the values for the 5th to 95th percentiles. D shows mean±s.e.m. *P<0.05 (one-way ANOVA with Tukey's post hoc test). a.u., arbitrary units. (E) Model. (a) PCM proteins are recruited to the centrosomes at late G2 phase. Molecular dynamics of CEP215 is upregulated for formation of compact, functional spindle poles at M phase. (b) In the absence of CEP215, PCM proteins are insufficiently recruited to the centrosomes, resulting in spindle defects. (c) PCM proteins are not properly recruited to the centrosomes when molecular dynamicity of CEP215 is reduced in mitotic centrosomes, resulting in spindle defects.

We initially found that the CCD-truncated mutants of CEP215 have reduced molecular mobilities in the cytoplasm. We next explored whether the molecular mobility of the CEP215 mutants is also reduced at the centrosomes. To test the hypothesis, we generated stable HeLa cells in which endogenous CEP215 was replaced with the FLAG–mCh–CEP215 mutants. Immunoblot analysis revealed that all the CEP215 mutants were expressed at comparable levels to the endogenous CEP215 (Fig. 6D), and their localizations were largely limited to the centrosomes throughout the cell cycle (Fig. S5B). We performed FRAP analyses of the FLAG–mCh–CEP215 mutants at the mitotic centrosomes, and revealed that the fluorescence signals of wild-type (WT) and BS1-truncated mutant of FLAG–mCh–CEP215 recovered efficiently in the mitotic centrosomes (Fig. 6E,F). However, those of the CCD-truncated mutants and PCNT-interaction-defective mutants of FLAG-mCh-CEP215 exhibited much reduced diffusive behaviors (Fig. 6E,F), suggesting that molecular dynamics of the CEP215 mutants are more or less identical in both the centrosomes and cytoplasm. Another possibility is that the truncated CEP215 mutant proteins are not efficiently recruited to mitotic centrosomes (see Fig. 7). In this case, the reduced FRAP activity might partly result from the low molecular dynamics of mCh–CEP215, which persists from interphase centrosomes.

We explored importance of CEP215 dynamics in spindle pole assembly. First, we determined centrosome levels of the CCD-truncated mutants of CEP215 at mitotic cells. The results revealed that spindle pole levels of both FLAG–mCh–CEP215^ΔCCD5^ and FLAG–CEP215^L1867S^ were significantly reduced, whereas those of FLAG–mCh–CEP215 and FLAG–CEP215^ΔBS1^ were unaffected (Fig. 7A,B). The spindle pole levels of endogenous PCNT were also reduced in FLAG–CEP215^L1867S^ mutant cells (Fig. 7A,C). We also determined centrosome areas of CEP215 and selected PCM proteins during the cell cycle. As previously shown (Lee and Rhee, 2011), the centrosome areas of CEP215 and PCNT reached a maximum at the end of G2 phase and then slightly reduced in mitotic cells, whereas those of γ-tubulin and CEP192 continuously increased until the M phase (Fig. 7D; Fig. S7). However, the same PCM proteins were not efficiently accumulated in cells with the replaced FLAG–mCh–CEP215^ΔCCD3^ and FLAG–CEP215^L1867S^ (Fig. 7D). These results suggest that CEP215 dynamicity shows a strong correlation with centrosome accumulation of PCM and its installation in mitotic cells.

**Fig. 8.**
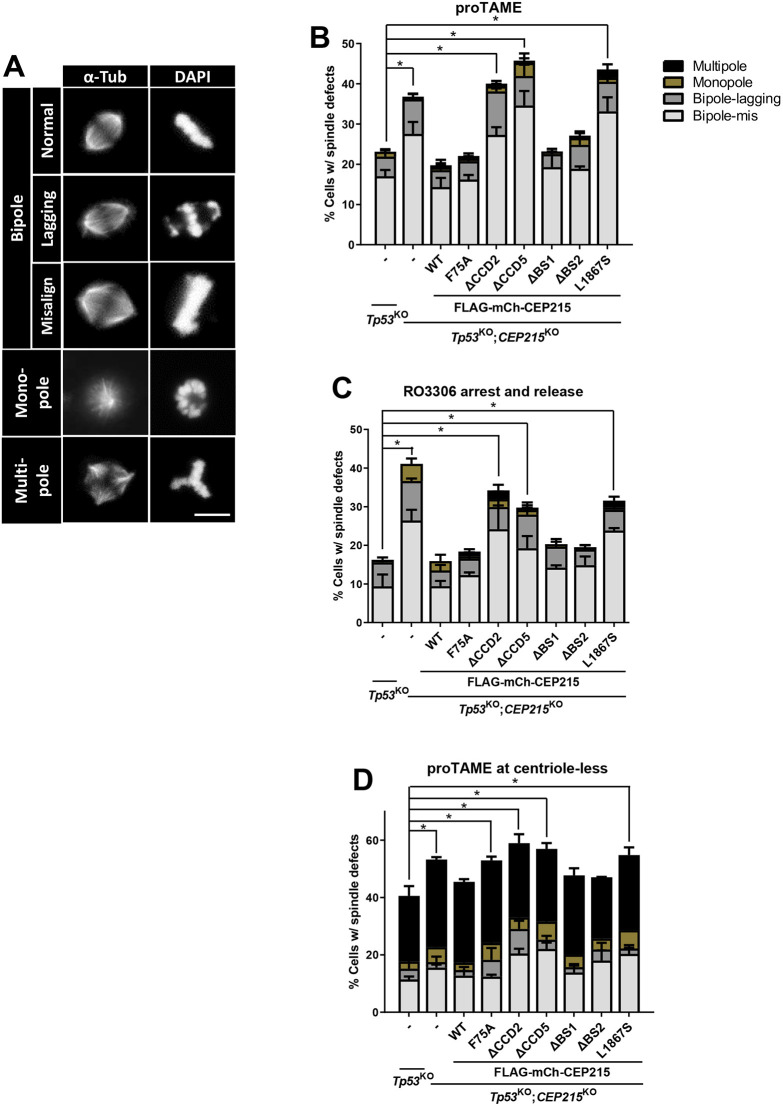
**Spindle defects in the FLAG–mCh–CEP215 mutant replacement cells.** (A) Representative images of the spindle defect phenotypes. Scale bar: 10 μm. (B) The FLAG–mCh–CEP215 mutant replacement HeLa cells were arrested at metaphase with the proTAME treatment. Representative images are shown in Fig. S8. (C) Metaphase cells were enriched with the RO3306 treatment and release method. (D) Cells were treated with centrinone B for 5 days to get rid of the centrioles. The cells were then treated with proTAME for metaphase arrest and their spindle morphology was determined. In B–D, the cells were immunostained with anti-α-tubulin antibody and their spindle phenotypes were determined. At least 50 cells per experimental group were counted in 3 independent experiments. Values are mean±s.e.m. *P<0.05 (one-way ANOVA with Tukey's post hoc test).

To determine the functional importance of CEP215 dynamics during mitosis, we examined the spindle formation activities of the HeLa cells where the endogenous CEP215 was replaced with ectopic FLAG–mCh–CEP215 variants. The CEP215 mutant-rescued cells were treated with proTAME for the metaphase arrest and co-immunostained with anti-α-tubulin, γ-tubulin and CEP215 antibodies to visualize mitotic spindles (Chinen et al., 2021). Spindle defects were classified as monopoles, multipoles, normal and abnormal bipoles with misaligned chromosomes within the spindles and with lagging chromosomes outside the spindles (Fig. 8A). As expected, the *p53*^KO^;*CEP215*^KO^ cells had a reduced rate of normal spindle formation and the ectopic expression of FLAG–mCh–CEP215^WT^ rescued cells from spindle defects (Fig. 8B; Fig. S8). In this condition, the FLAG–mCh–CEP215 CCD truncated mutants (FLAG–mCh–CEP215^ΔCCD2^ and FLAG–mCh–CEP215^ΔCCD5^) and FLAG–CEP215^L1867S^ failed to rescue the spindle defects, whereas other truncated mutants, at the CEP192- and HSET-binding sites (FLAG–mCh–CEP215^ΔBS1^ and FLAG–mCh–CEP215^ΔBS2^), and FLAG–mCh–CEP215^F75A^ exhibited a fully recovered phenotype (Fig. 8B; Fig. S8). We also assessed metaphase cells obtained through a thymidine-RO3306 release method, and observed mitotic spindles in the rescued cells. The results are quite similar to those with the proTAME-arrested cells (Fig. 8C). In summary, mitotic spindles were not accurately formed in the HeLa cells whose CEP215 was replaced with the dynamicity-reduced mutants.

When centrosomes become spindle poles in mitosis, they have to accumulate a vast amount of PCM proteins for robust microtubule organization activities. The major constituents of the mitotic PCM are CEP215 and PCNT. We hypothesized that dynamicity of CEP215 and possibly PCNT might be upregulated for the initiation and maintenance of mitotic PCM, and tested this hypothesis using centriole-less centrosomes, in which mitotic PCM alone functions as spindle poles (Watanabe et al., 2020; Chinen et al., 2021). The cells were treated with centrinone, a PLK4 inhibitor, for 5 days to remove centrioles from the cells, and then with proTAME for metaphase arrest. We observed that over 40% of the cells had abnormal spindles, probably because they lacked centrioles in the *p53*^KO^ background (Fig. 8D; Watanabe et al., 2020). The spindle defect rates increased over 50% with additional deletion of CEP215 and were rescued upon expression of FLAG–CEP215^WT^ (Fig. 8D). However, FLAG–CEP215^ΔCCD2^, FLAG–CEP215^ΔCCD5^ and FLAG–CEP215^L1867S^ were not able to rescue the spindle defects significantly (Fig. 8D). These results further suggest that CEP215 should dynamically interact with PCM proteins for the proper formation of mitotic spindles.

## DISCUSSION

In this study, we investigated molecular dynamics of CEP215 in human cells. Our FRAP analysis revealed that CEP215 exhibits a dynamically suppressed, solid-like state in interphase centrosomes, yet it transitions to a more dynamic state in mitotic centrosomes. This finding suggests that a physical property of CEP215 can interchange, depending on the cell cycle stage. PCM in interphase centrosomes is concentrically arranged around the centrioles (Lawo et al., 2012; Mennella et al., 2012). In this configuration, CEP215 likely maintains a low molecular dynamicity through high-affinity interactions to other centrosome components. Conversely, in mitotic centrosomes, additional PCM proteins accumulate with a less organized structure, facilitating a robust microtubule organization for mitotic spindle formation (Lawo et al., 2012; Conduit et al., 2014). Under these conditions, CEP215 appears to have a highly mobile state. Our results align with previous research indicating that the liquid-like state of SPD-5 can transform into a more viscous gel or solid-like state over a time (Woodruff et al., 2017).

It remains largely unclear how the molecular dynamicity of CEP215 is regulated during the cell cycle. In this work, we found a hint that CCDs are important for CEP215 dynamics, given that their disruption limited diffusive mobility of the CEP215 protein. CEP215 is a multivalent protein with multiple CCDs involved in homotypic interactions, as shown in *C. elegans* SPD-5 (Ohta et al., 2021; Nakajo et al., 2022), *Drosophila* Cnn (Feng et al., 2017; Tovey et al., 2021) and human CEP215 (Yang et al., 2023). Our co-immunoprecipitation results also indicate homotypic interactions of CEP215, even if the LZ–CM2 interaction is not confirmed. Furthermore, we found that CEP215 CCDs play a crucial role in spindle pole assembly in mitotic cells. We propose that diffusive mobility of CEP215 is controlled by both homotypic and heterotypic interactions among CCDs, especially at mitotic spindle poles. Recent reports indicate that the multimerization property of CCDs is sufficient to regulate the molecular dynamicity of proteins, possibly through phase separation (Ramirez et al., 2024). Indeed, the importance of CCDs in phase separation has also also demonstrated in TAZ (also known as WWTR1), a downstream transcription factor of the Hippo pathway, where CCD-truncated TAZ mutants do not undergo phase separation nor induce target gene transcription (Lu et al., 2020). PLK1 is known to regulate the protein structure of SPD-5, allowing new homotypic and heterotypic interactions among CCDs (Rios et al., 2024). It would be interesting to examine whether PLK1 phosphorylation also regulates diffusive mobility of CEP215 by controlling CCD-mediated protein–protein interactions. This line of investigation could reveal further regulatory mechanisms underlying the dynamic structural adaptations of centrosome components during the cell cycle.

Sequence-specific heterotypic interactions with other proteins can thermodynamically modulate protein phase behaviors (Lee et al., 2024b). CEP215 is known to interact with various centrosome proteins, including dynein–dynactin (Lee and Rhee, 2010), PCNT (Buchman et al., 2010; Kim and Rhee, 2014; Wang et al., 2010), HSET (Chavali et al., 2016), EB1 (also known as MAPRE1) (Fong et al., 2009) and CEP192 (Kuriyama and Fisher, 2020). However, it remains unclear whether the molecular dynamics of CEP215 is regulated by these specific interactions. Our study indicates that specific interactions with HSET and possibly CEP192 might not be essential for diffusive ability of CEP215. By contrast, we clearly demonstrated that the interaction with PCNT is crucial for diffusive mobility of CEP215. We imagine that the CEP215–PCNT complex might maintain a flexible configuration, so that it does not form crystals *in vitro* even at a high concentration (Fig. 4C).

FRAP analysis revealed reduced dynamics of the mutant proteins both in the cytoplasm and at the centrosome. Furthermore, mitotic spindle defects were observed in cells when endogenous CEP215 was replaced with mutant forms (Fig. 8). Based on these observations, we propose that facilitated dynamicity of CEP215 might be required for PCM recruitment for mitotic spindle formation (Fig. 7E). The reduced dynamics of CEP215 contribute to the mitotic spindle defects, possibly due to inefficient recruitment of CEP215 to the mitotic centrosomes (Fig. 7E). It remains to be investigated how dynamicity of CEP215 is regulated during the cell cycle stages. Furthermore, it would be important to investigate the biophysical behaviors of other centrosome proteins in interphase and mitotic centrosomes.

## MATERIALS AND METHODS

### Antibodies and plasmids

For immunostaining analyses, we used rabbit antibodies against CEP215/CDK5RAP2 (06-1398; Merck Millipore; 1:100), PCNT (Lee and Rhee, 2011), CEP192 (A302-324A; Bethyl Laboratory; 1:100), α-tubulin (ab18251; Abcam), γ-tubulin (ab11317; Abcam; 1:100); mouse antibodies against centrin2 (04-1624; Merck Millipore), α-tubulin (T6199; Sigma; 1:100), FLAG (F3165; Sigma; 1:100); and goat anti-FLAG (ab1257; Abcam; 1:100) antibodies. Alexa Fluor 488- and 594-conjugated secondary antibodies (Invitrogen; 1:100) were also used. For immunoblot analyses, we used CEP215 (06-1398, Merck; 1:1000), PCNT (Lee and Rhee, 2011; 1:1000), γ-tubulin (ab11317, Abcam; 1:1000); CEP192 (A302-324A, Bethyl; 1:1000), FLAG (F3165, Sigma; 1:2000) and GAPDH (AM4300, Ambion; 1:10,000) antibodies. Anti-mouse IgG-HRP (A9044; Sigma-Aldrich; 1:10,000) and anti-rabbit IgG (AP132P; Millipore; 1:10,000) were used as secondary antibodies.

The *CEP215* full length and mutant genes were subcloned into the *pCDNA5* vector (V1033-20; Gibco) tagged with *FLAG*, *mCherry* and *CRY2* sequences. *MBP-CEP215*^1661-1893^ was subcloned into the *pPROEX* vector (V003918; NovoPro). *MBP-CEP215*^1814-1877^ and *MBP-PCNT*^2388-2420^ were subcloned into the *pET Duet-1* vector (71146-3; Novagen) and transformed into *E. coli* Rosetta (DE3) cells. For *CEP215* deletion mutants, we generated ΔCM2 (Δ1726-1893), ΔCCD1 (Δ216-243), ΔCCD2 (Δ279-311), ΔCCD3 (Δ1195-1231), ΔCCD4 (Δ1396-1425), ΔCCD5 (Δ1483-1510), ΔBS1 (Δ363-390), ΔBS2 (Δ434-590) and ΔBS3 (Δ730-760). We also prepared a GFP-linked centrin-2 expression vector (pGFP-centrin-2).

### Cell culture, transfections and drug treatment

HeLa and 293T cells (CRL-3276; ATCC) were cultured in DMEM (Welgene, LM 001–05) supplemented with 10% fetal bovine serum (Welgene, S101-01) and antibiotics (Invivogen, ANT-MPT) at 37°C.

For transient expression of the ectopic proteins, HeLa cells were transfected with 5 μg of the expression vectors using Lipofectamine 3000 (Invitrogen) according to the manufacturer's instruction. For the immunoprecipitation assays, the plasmids were transiently transfected into 293T cells using the polyethyleneimine method. For establishment of stable cell lines, 2.4×10^5^ HeLa Flp-In TREX cells were seeded on a 60-mm dish and 4 μg of the expression vectors was transfected using Lipofectamine 3000 (Invitrogen). Two days later, the cells were treated with hygromycin (0.4 mg/ml; 10687010; Thermo Fisher Scientific) for 3 weeks. To induce ectopic proteins, stable cell lines were treated with doxycycline (1 mg/ml; D5207; Sigma) for 38 h.

To obtain metaphase populations, the cells were treated with 20 μM of proTAME (#25835, Cayman) for 4 h. To obtain M phase-enriched populations, the cells were treated with 2 mM thymidine (Sigma, T9250) for 24 h, washed and treated with 5 μM RO3306 (ALX-270-463; Enzo life sciences) for 8 h, washed out with warmed DMEM and cultured for 40 min. To eliminate centrioles, the cells were treated with 125 nM of centrinone B (a kind gift from Karen Oegema, University of California, San Diego, USA) for 5 days.

### Live imaging and FRAP analysis

Live-cell imaging was performed using a Nikon 60× oil immersion objective (NA 1.4; Nikon, MXA22168) on a Nikon A1 laser scanning confocal microscope equipped with a CO_2_ microscope stage incubator under 5% CO_2_ at 37°C. Fluorescence recovery after photobleaching (FRAP) experiments were performed with a laser at a wavelength of 561 nm using the region of interest (ROI) stimulation function in the Nikon A1 confocal microscope. After taking 5 initial images with no delay, bleaching was performed, and images were acquired at 10-s intervals. The *z*-plane of the image was adjusted by tracking GFP–centrin-2-positive centrosomes in the cell.

### Immunoblotting

Cells were lysed with the RIPA buffer (50 mM Tris-HCl, pH 8.0, 150 mM NaCl 1% Triton X-100, 0.5% C_24_H_39_NaO_4_, and 0.1% SDS) containing a protease inhibitor cocktail (Sigma-Aldrich, P8340) and centrifuged at 10,000 ***g*** for 10 min at 4°C. The supernatants were mixed with 4×SDS sample buffer (250 mM Tris-HCl pH 6.8, 8% SDS, 40% glycerol and 0.04% Bromophenol Blue) and 10 mM DTT (Amresco, 0281-25G), boiled for 5 min, electrophoresed and transferred onto Protran BA85 nitrocellulose membranes (GE Healthcare Life Sciences, 10401196). The membranes were blocked with blocking solution (5% nonfat milk or bovine serum albumin in 0.1% Tween 20 in TBS) for 1–2 h, incubated with primary antibodies diluted in blocking solution for 24 h at 4°C, washed three times with 0.1% Tween 20 in TBS (TBST), then incubated with secondary antibodies in blocking solution for 30 min and washed again. To detect the signals of secondary antibodies, ECL reagent (ABfrontier, LF-QC0101) and X-ray films (Agfa, CP-BU NEW) were used. Uncropped images of western blots from this paper are shown in Fig. S9.

### Immunoprecipitation

293T cells were transfected with the expression vectors using polyethylenimine. After transfection, the cells were lysed with a lysis buffer (50 mM Tris-HCl, pH 8.0, 5 mM EDTA, 150 mM NaCl, 0.5% Triton X-100, protease inhibitors, 0.5 mM PMSF, and 1 mM DTT) for 20 min on ice. After centrifugation at 10,000 ***g*** for 20 min, the supernatants were incubated with anti-FLAG antibody-tagged protein-A-Sepharose (GE Healthcare) for 1 h. The beads were washed three times with the lysis buffer and subjected to immunoblot analyses. All the procedures were performed at 4°C.

### Immunocytochemistry and image processing

For immunocytochemistry, HeLa cells were cultured on 12-mm coverslips. The cells were fixed with ice-cold 100% methanol for 10 min, washed with PBS three times and permeabilized with PBS with 0.1% Triton X-100 (PBST) for 10 min. Cells were then blocked with 3% BSA in 0.3% PBST for 30 min. Primary antibodies in 3% BSA was loaded on each cover slips and incubated for 1 h. Then, cells were washed three times with PBST and incubated with secondary antibodies conjugated to Alexa Fluor 488, 594 and 647-nm (Invitrogen) in blocking solution for 30 min. After incubation, coverslips were washed twice with PBST, incubated with DAPI solution for 2 min and washed twice with PBST. The coverslips were mounted on a glass slide with ProLong Gold antifade reagent (Invitrogen, P36930). Observation of the samples was done using a fluorescence microscope (Olympus IX51) equipped with a CCD (Qicam fast 1394, Qimaging) camera. The image analysis was undertaken using Image Pro 5.0 software (Media Cybernetics). Inset images were enlarged four times in Photoshop CS6 (Adobe) using option of bicubic interpolation. In the cases of live imaging capture, Image 5D (Fiji; https://imagej.net/plugins/image5d), was used.

### Quantification of arbitrary expression level

HeLa cells were transiently transfected with the CEP215 expression vectors. After 48 h, the cells were light-protected by wrapping with an aluminum foil for 10 min and activated under a 488 nm light for indicated time periods. After activation, cells were fixed with 100% cold methanol, immunostained with the FLAG antibody, and observed using fluorescence microscope (Olympus IX51) equipped with a CCD (Qicam fast 1394, Qimaging) camera. Intensities of individual cells were determined using ImageJ program with the same size of region of interest.

### Protein expression and purification

All bacterially expressed CEP215 and PCNT proteins in this study were tagged with MBP to enhance expression and solubility. Bacterial cells were grown at 37°C until the optical density at 600 nm (OD600) reached 0.7, and protein expression was induced with 0.3 mM IPTG at 20°C overnight. After harvesting, cell pellets were resuspended in lysis buffer (20 mM HEPES pH 7.5, 500 mM NaCl, 1 mM TCEP). Cells were lysed using an Emulsiflex C3 (Avestin) in the presence of 0.1 mM PMSF and DNase I (Roche). After centrifugation at 13,000 ***g*** for 30 min, cleared lysates were loaded onto an amylose column pre-equilibrated with lysis buffer. Bound proteins were eluted with a buffer containing 20 mM maltose after extensive washing with lysis buffer. Eluted protein was concentrated and further purified by size exclusion chromatography (Superdex 200 10/300 GL, GE Healthcare) using a buffer consisting of 25 mM HEPES pH 7.5, 500 mM NaCl, and 1 mM TCEP.

Bacterial cells were lysed in PBS buffer with an Emulsiflex C3 (Avestin) in the presence of 0.1 mM PMSF and DNase I (Roche). After centrifugation at 13,000 ***g*** for 30 min, cleared lysates were loaded onto an amylose column pre-equilibrated with lysis buffer. Bound proteins were eluted with a 20 mM maltose buffer after extensive column washing with lysis buffer. The MBP tag was cleaved by TEV protease in cleavage buffer (20 mM Tris-HCl pH 8.0, 100 mM NaCl, 2 mM DTT) and removed by anion exchange chromatography (Hitrap Q HP, GE Healthcare). Finally, the protein was further purified by size exclusion chromatography (Superdex 200 10/300 GL, GE Healthcare) using a buffer consisting of 25 mM HEPES pH 7.5, 500 mM NaCl and 1 mM TCEP.

Size exclusion chromatography coupled with multi-angle light scattering For determination of molecular mass, size exclusion chromatography coupled to multi-angle light scattering (SEC-MALS) was used. Each purified protein sample was concentrated to 1 to 5 mg/ml and applied to a Superdex 200 Increase 5/150 GL column (GE Healthcare) connected to MALS-UV-RI (refractive index) detectors (Wyatt TREOS). Protein concentration and the observed scattering signal were used to calculate the molecular mass with Zimm fit method (*d*n/*d*c value of 0.1850 ml/g) as implemented in Wyatt's ASTRA 7.14 software.

The heterotetrameric CEP215–PCNT complex model was computed using AlphaFold3 (Abramson et al., 2024), using the sequences of human CEP215 (residues 1808–1877) and PCNT (residues 2388–2412), with two copies of each chain and one zinc ion as input.

### Isothermal titration calorimetry

The ITC experiment was performed using a MicroCal PEAQ-ITC (Malvern Instruments Ltd., UK). 25 µM of MBP–CEP215^1661-1893^ protein was loaded into the cells and titrated with 250 µM of PCNT^2350-2450^ at 25°C. After 29 injections, titration curves and thermodynamic parameters were calculated using the MicroCal PEAQ-ITC Analysis Software.

## Supplementary Material



10.1242/joces.263542_sup1 Supplementary information
