# Supplementary material for: Enhancement of CEP215 dynamics for spindle pole assembly during mitosis
Source: J Cell Sci. 2025 May 21;138(10):jcs263542. doi: 10.1242/jcs.263542 (PMC12148038; doi:10.1242/jcs.263542)
Supplement: Supplementary information [file joces-138-263542-s1.pdf]

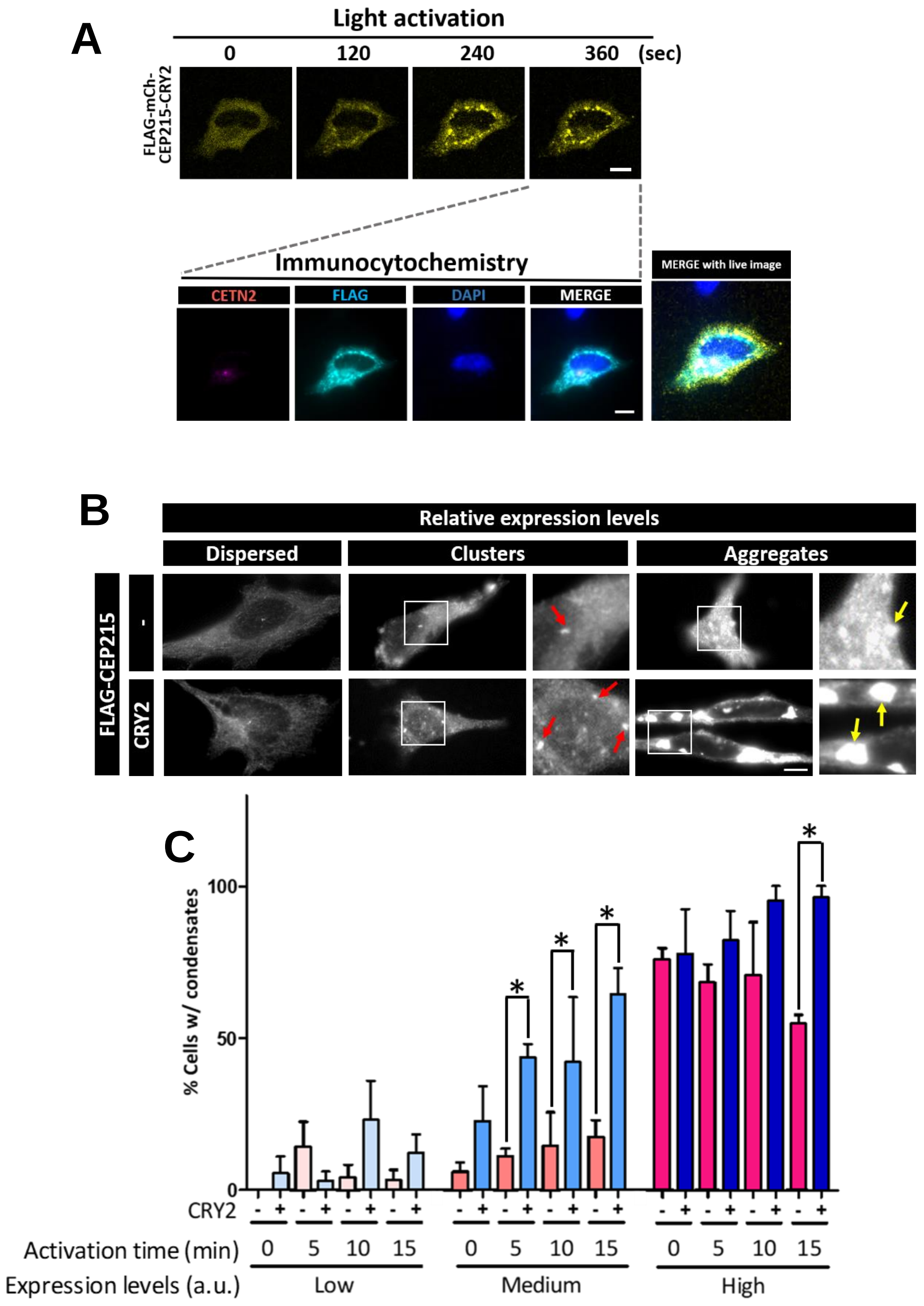

Fig. S1. Immunostaining analysis for detection of cytoplasmic condensates of FLAG-mCh-CEP215-CRY2 (A) The FLAG-mCh-CEP215-CRY2-expressing HeLa cells were light-activated to form opto-droplets (upper). The same cell was coimmunostained with centrin-2 (magenta) and FLAG (cyan) antibodies. DNA was stained with DAPI (blue). The immunostaining pattern of FLAG was overlaid with the live fluorescent image (yellow) of FLAG-mCh-CEP215-CRY2. (B) Representative images of dispersed, clustered (red arrows) and aggregates (yellow arrows) of FLAG-CEP215 and FLAG-CEP215-CRY2. (A, B) Scale bars, 10 µm. (C) The number of cells with cytoplasmic clusters was counted after blue light activation for indicated time periods. At least 90 cells per experimental group were counted in 3 independent experiments. Values are means with standard deviations. Statistical significance was determined using one-way ANOVA with Tukey's post hoc test. *, $P<0.05$.

Journal of Cell Science • Supplementary information

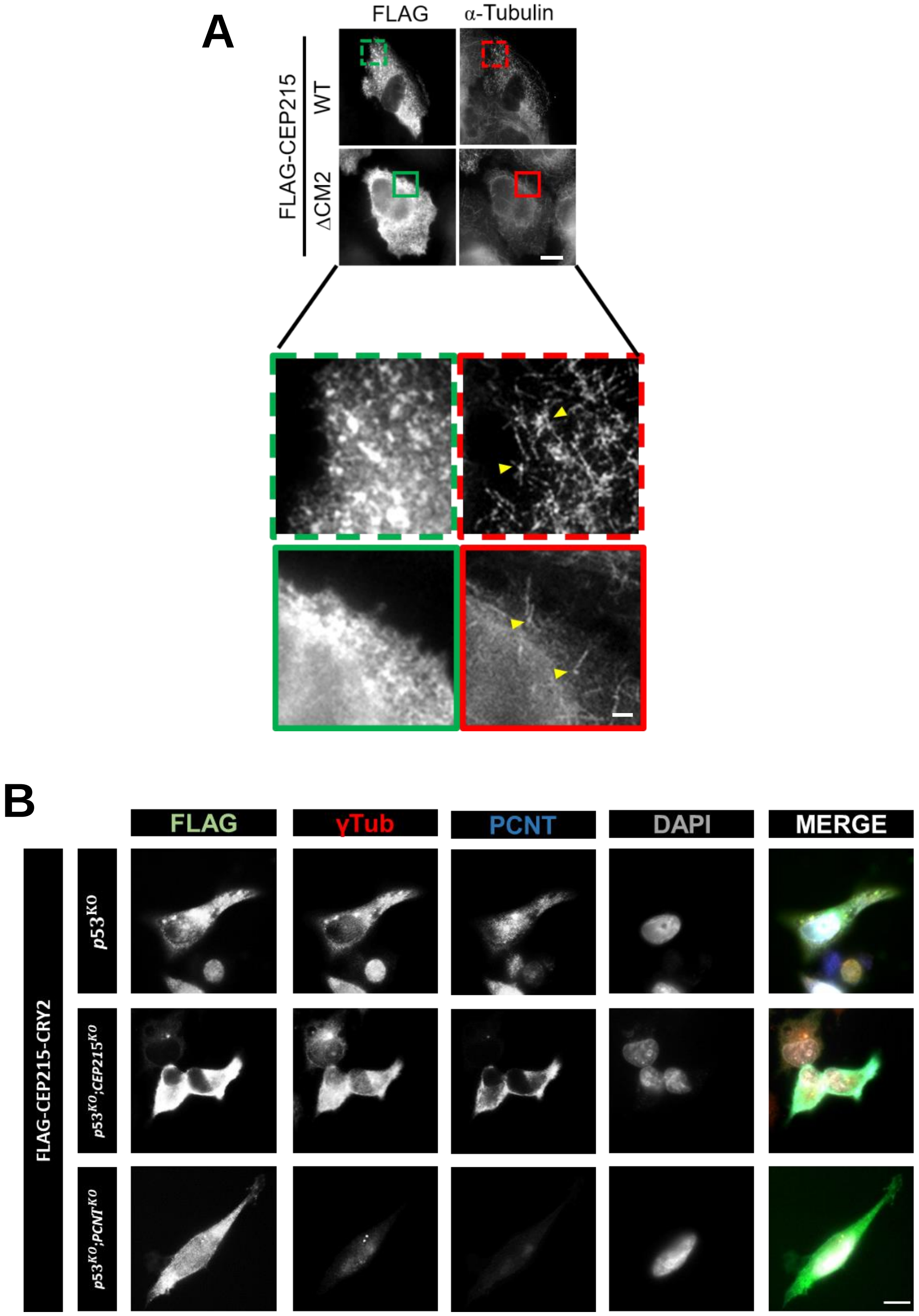

Fig. S2. (A) Microtubule regrowth assays The wild-type and ΔCM2 FLAG-mCh-CEP215-CRY2-expressing HeLa cells were treated nocodazole (1.65 μM) for 1 h to induce microtubule repolymerization. The cells were then light-activated and incubated with pre-warmed fresh medium for 10 min, and immediately fixed for immunostaining with FLAG and α-tubulin antibodies. Enlarged images are shown below. Arrowheads indicates microtubules grown from the cytoplasmic clusters of FLAG-mCh-CEP215-CRY2. Scale bars, 1 and 10 μm. **(B) Representative images of Fig. 3E.** FLAG-CEP215-CRY2 was transiently expressed in the *p53*^{KO}, *p53*^{KO};*CEP215*^{KO} and *p53*^{KO};*PCNT*^{KO} cells. The cells were light-activated and coimmunostained with FLAG, γ-tubulin and PCNT antibodies. DNA was stained with DAPI. Scale bar, 10 μm.

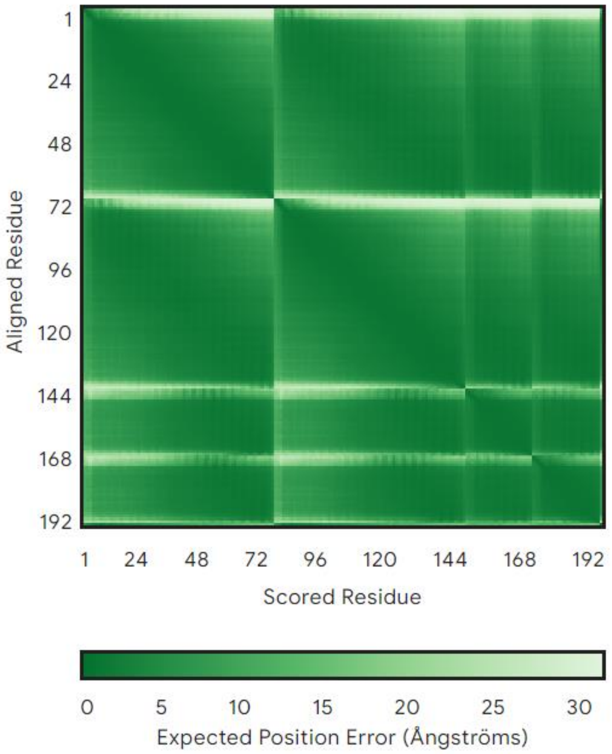

Fig. S3. Expected position error for CEP215-PCNT tetramer from AlphaFold3 prediction This heatmap visualizes the expected position error in the AlphaFold3 model of the CEP215$^{1808-1877}$–PCNT$^{2388-2412}$ tetramer. Residues 1-71 and 72-142 correspond to CEP215, while residues 143-167 and 168-192 correspond to PCNT. The color scale ranges from 0 Å (high accuracy) to 30 Å (low accuracy), with darker regions indicating more accurate structural predictions and lighter regions reflecting areas of greater uncertainty.

Journal of Cell Science • Supplementary information

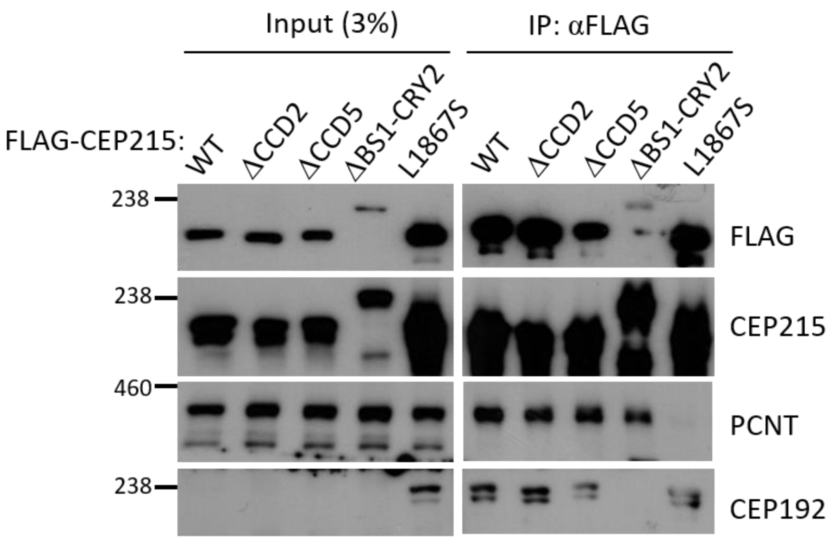

Fig. S4. Coimmunoprecipitation analysis of FLAG-mCh-CEP215 mutants with endogenous PCM proteins The FLAG-CEP215 proteins (WT, ΔCCD2, ΔCCD5, ΔBS1-CRY2, and L1867S) were expressed in 293T cells. Twenty-four hours later, the cells were subjected to immunoprecipitation with the FLAG antibody, followed by immunoblot analyses with FLAG, CEP215, PCNT, and CEP192 antibodies.

Journal of Cell Science • Supplementary information

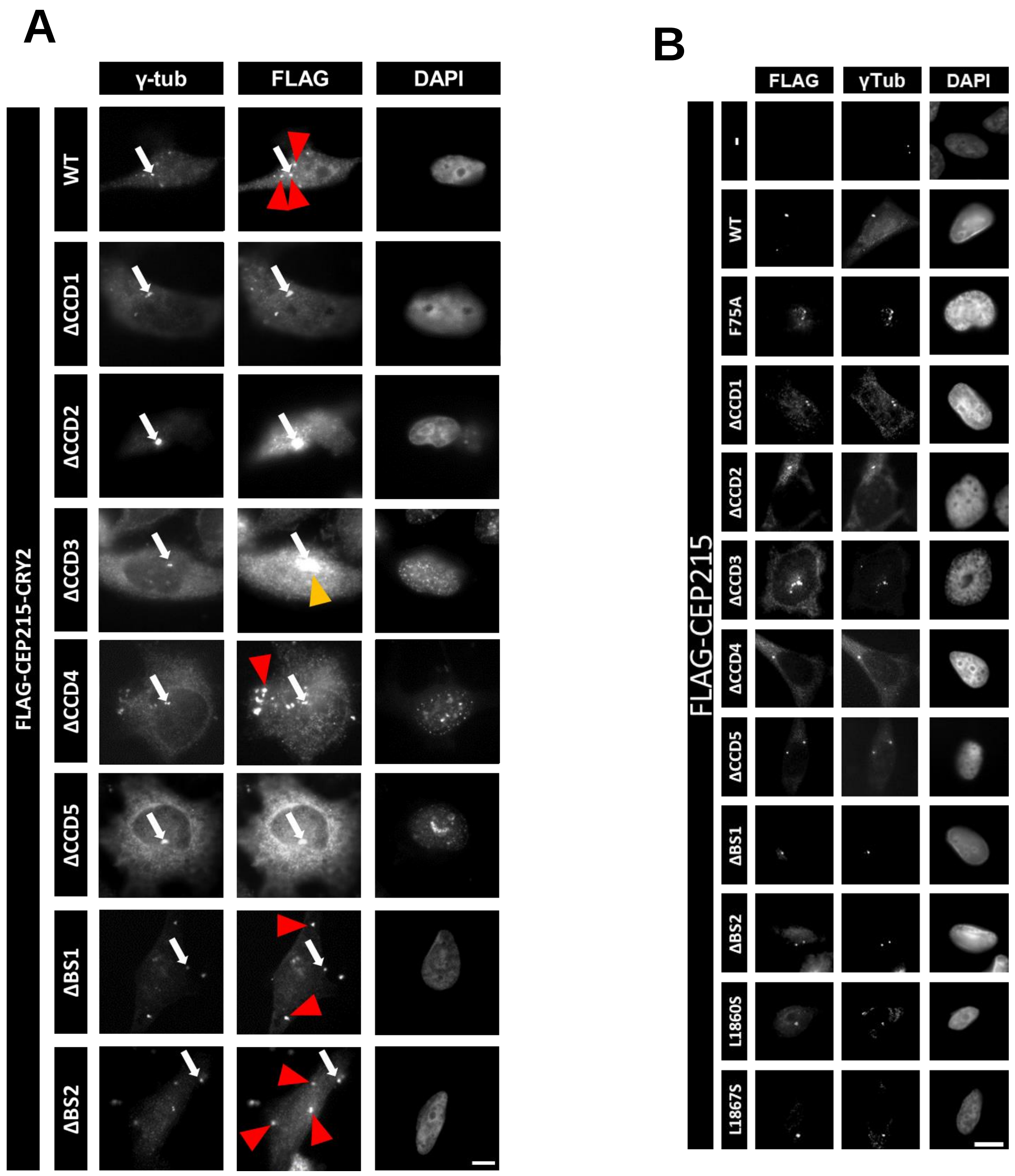

Fig. S5. (A) Reduction of the cytoplasmic cluster formation activity in FLAG-mCh-CEP215-CRY2-truncated mutant-expressing cells Indicated truncated mutants of FLAG-mCh-CEP215-CRY2 were transiently expressed in HeLa cells, light-activated, and coimmunostained with γ-tubulin and FLAG antibodies. Cytoplasmic clusters (red arrowheads) and aggregates (yellow arrowheads) of FLAG-mCh-CEP215-CRY2 were indicated. Arrows indicate the centrosomes. **(B) Centrosome localization of the FLAG-mCh-CEP215 mutant proteins** FLAG-mCh-CEP215-expressing stable cells at interphase were coimmunostained with FLAG and γ-tubulin antibodies.(A, B) Scale bars, 10 μm.

Journal of Cell Science • Supplementary information

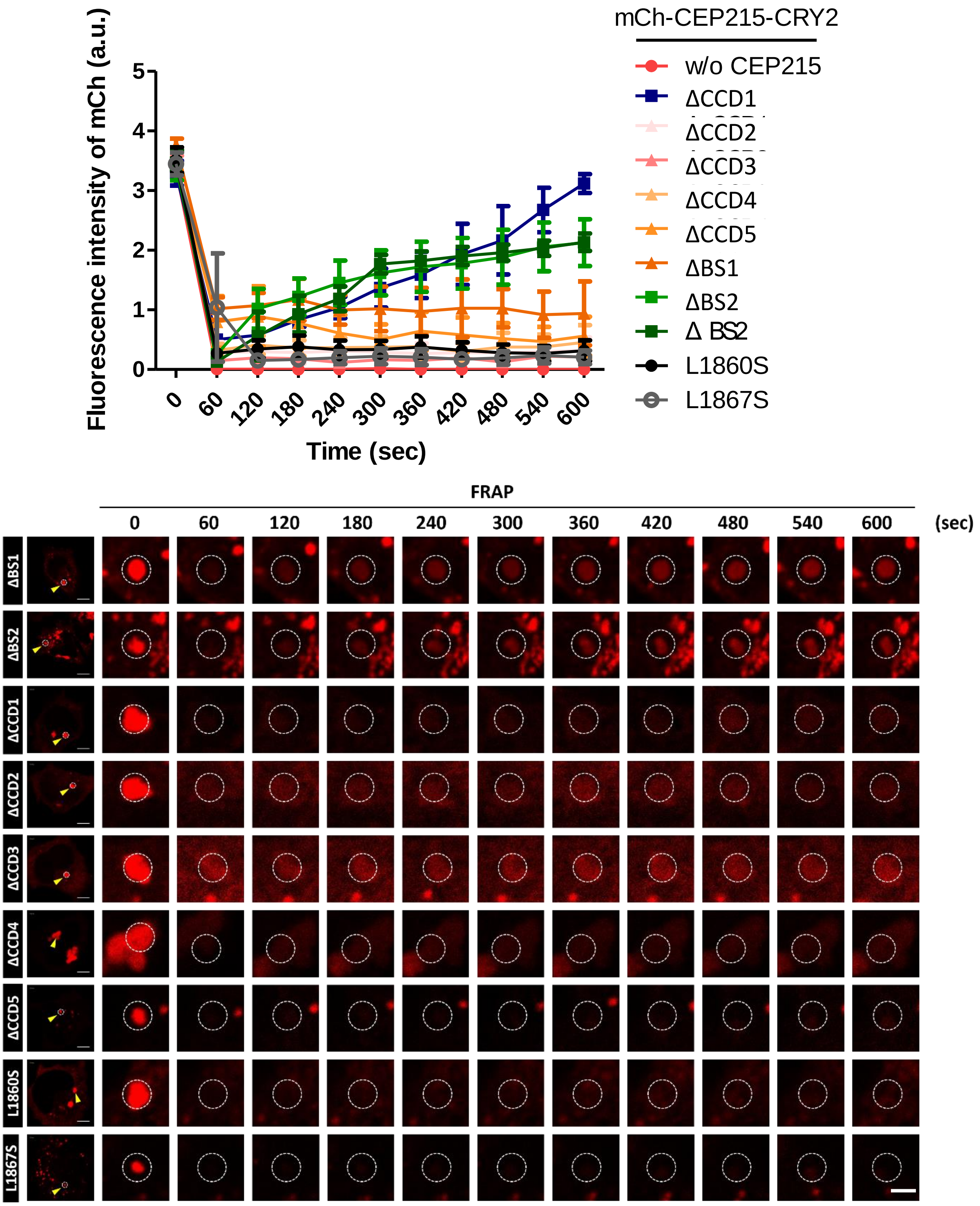

Fig. S6. FRAP analysis of the cytoplasmic clusters in mCh-CEP215-CRY2 mutant-expressing cells FRAP signals of the indicated mCh-CEP215-CRY2 mutant proteins at the cytoplasm were measured for up to 600 sec. At least 30 cells per experimental group were counted in 3 independent experiments. Values are means with standard deviations. Scale bar, 1 μm.

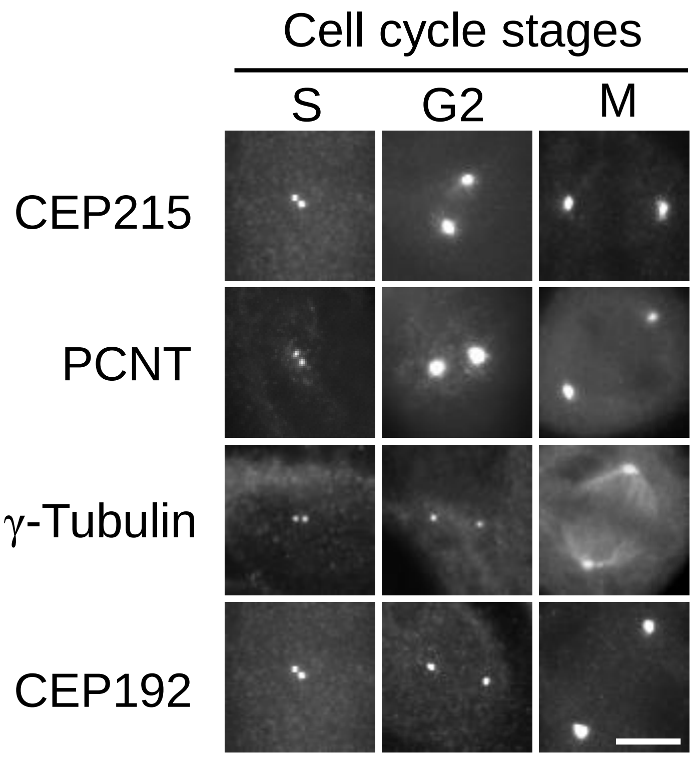

Fig. S7. Immunostaining analyses of PCM proteins during the cell cycle Cell cycle of the HeLa cells were synchronized with the double thymidine block and release. The S, G2 and M phase cells were coimmunostained with the CEP215, PCNT, γ-tubulin and CEP192 antibodies. The areas of specific signals were analyzed with the ImageJ program in Fig. 7D. Scale bar, 10 µm.

Journal of Cell Science • Supplementary information

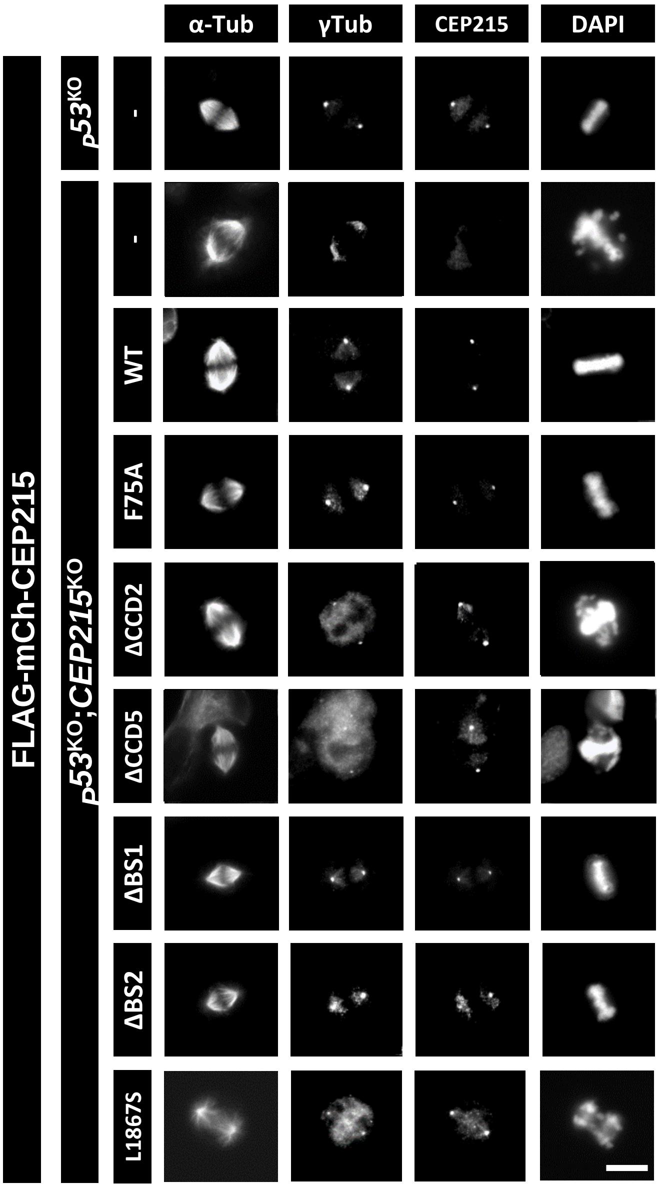

Fig. S8. Spindle defects in the *CEP215*KO cells rescued with the FLAG-mCh-CEP215 mutants Representative images of Fig 8B. The FLAG-mCh-CEP215 mutant-rescued HeLa cells were treated with proTAME to arrest the cell cycle at metaphase and coimmunostained with α-tubulin, γ-tubulin and CEP215 antibodies. Scale bar, 10 μm.

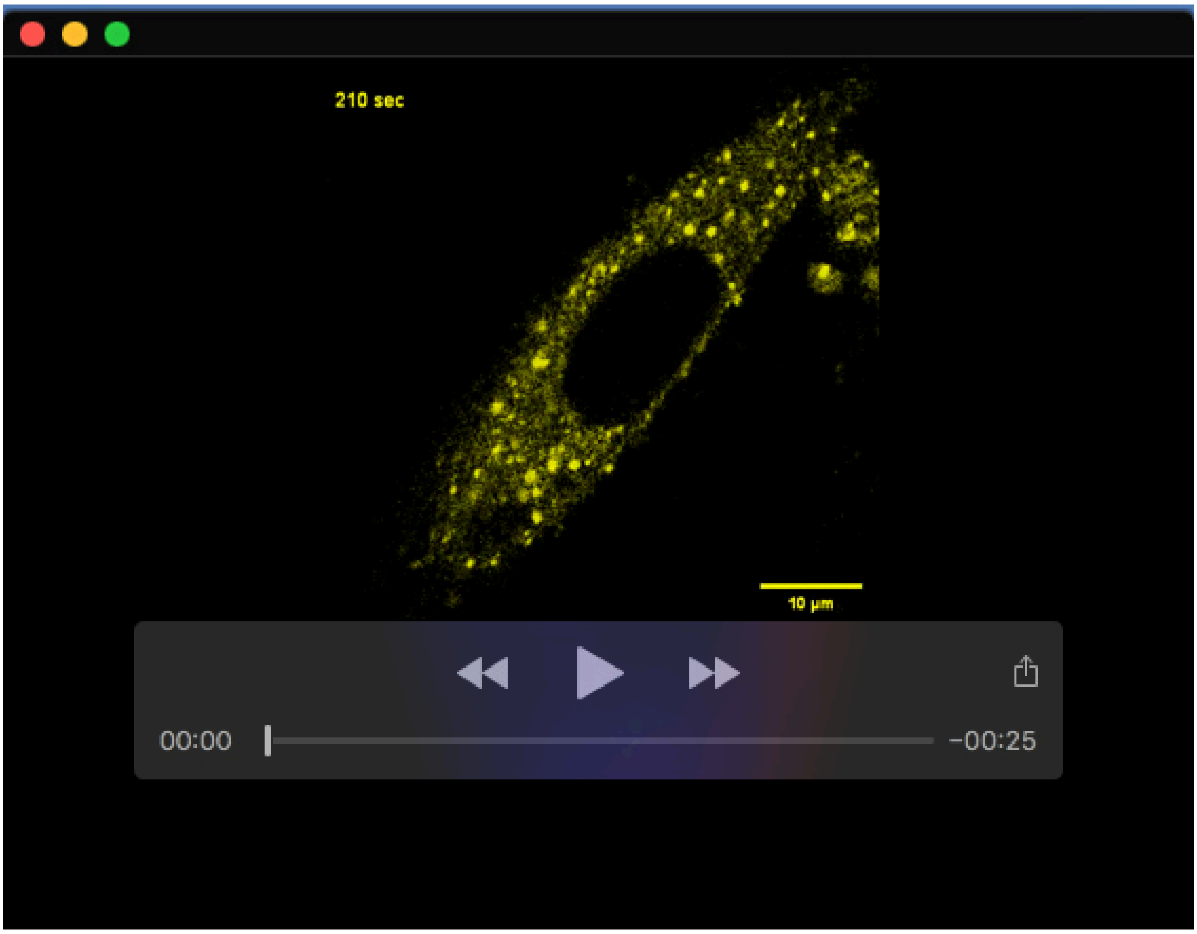

Movie 1. Clustering of mCh-CEP215-CRY2 after light activation. mCh-CEP215-CRY2 was transiently expressed in HeLa cells and light-activated for up to 140 sec, as shown in Fig. 2A. Scale bar, 10 µm.

Journal of Cell Science • Supplementary information

Fig. 1A

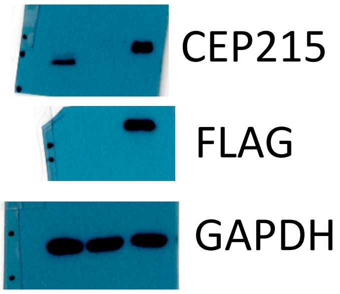

Fig. 6D

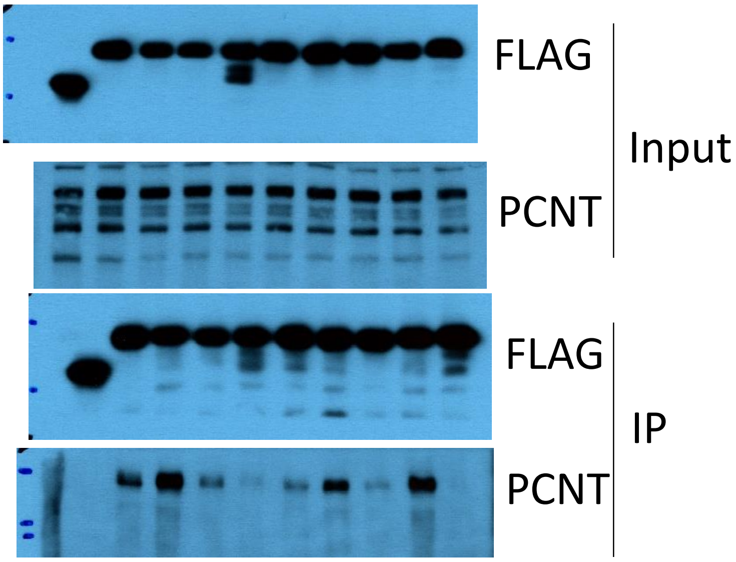

Fig. 5E

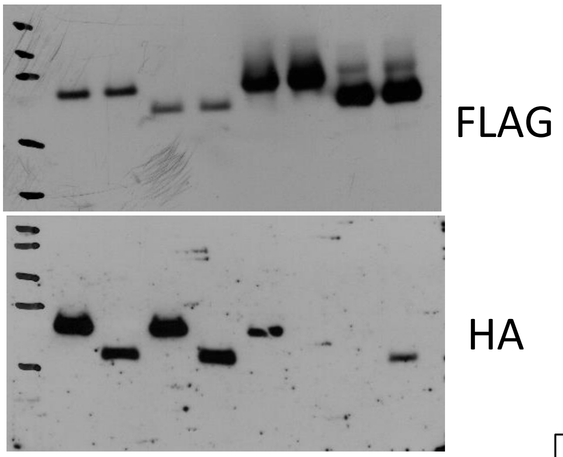

Fig. 6D

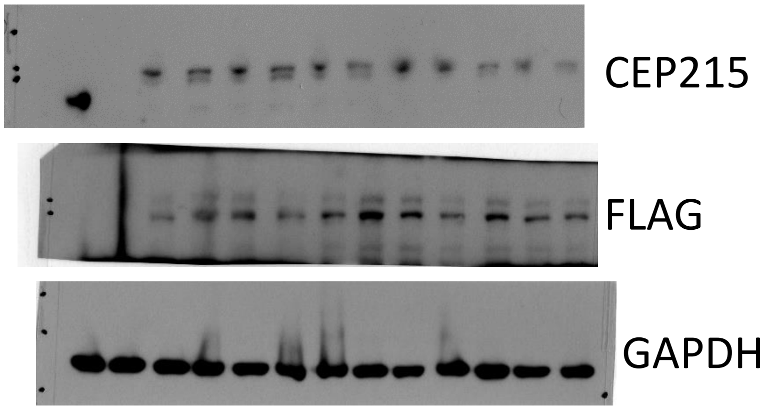

Fig. S4

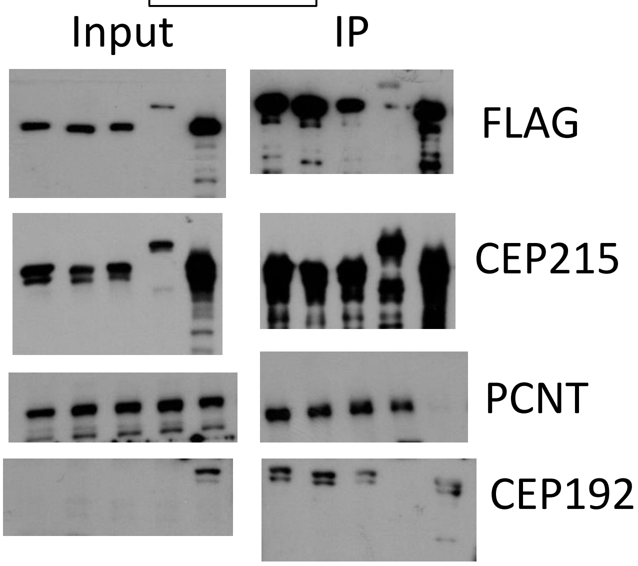

Fig. S9. Blot Transparency

Journal of Cell Science • Supplementary information
