# Supplementary material for: Enhancement of CEP215 dynamics for spindle pole assembly during mitosis
Source: J Cell Sci. 2025 May 21;138(10):jcs263542. doi: 10.1242/jcs.263542 (PMC12148038; doi:10.1242/jcs.263542)
Supplement: Supplementary file 1 [file joces-138-263542_review_history.pdf]

Enhancement of CEP215 dynamics for spindle pole assembly during mitosis

Hyunjeong Yoo, Taehyun Kim, Sungjin Ryu, Donghee Ko, Jeesoo Kim, Hee-Jung Choi, Yongdae Shin and Kunsoo Rhee
DOI: 10.1242/jcs.263542

Editor: Renata Basto

Review timeline

Original submission:	15 September 2024
Editorial decision:	8 October 2024
First revision received:	4 February 2025
Editorial decision:	3 March 2025
Second revision received:	31 March 2025
Editorial decision:	7 April 2025
Third revision received:	10 April 2025
Accepted:	15 April 2025

Original submission

First decision letter

MS ID#: jcs.263542

MS TITLE: Phase separation of CEP215 is essential for spindle formation during mitosis

AUTHORS: Hyunjeong Yoo; Taehyun Kim; Donghee Ko; Jeesoo Kim; Hee-Jung Choi; Yongdae Shin; Kunsoo Rhee

ARTICLE TYPE: Research Article

Dear Dr Rhee,

We have now reached a decision on the above manuscript.

As you will see, the reviewers raise a number of substantial criticisms that prevent me from accepting the paper at this stage. They suggest, however, that a revised version might prove acceptable, if you can address their concerns. If you think that you can deal satisfactorily with the criticisms on revision, I would be pleased to see a revised manuscript. We would then return it to the reviewers.

Reviewer 1

Advance summary and potential significance to field

Yoo et al. seek to reveal how the human centrosome assembles at the molecular level. They focus on assembly of the pericentriolar material (PCM), which comprises the bulk of the centrosome and serves as a scaffold to recruit microtubule nucleating and anchoring factors. Much of the mechanistic insight into PCM assembly has been determined through biochemical studies in worms

© 2025. Published by The Company of Biologists under the terms of the Creative Commons Attribution License (https://creativecommons.org/licenses/by/4.0/).

and flies, and it is not clear if those principles apply in distantly related mammals. Thus, the authors are addressing an important topic.

The authors claim that PCM assembles through liquid-liquid phase separation of Cep215. They further identify several domains (two coiled-coil regions) and select amino acids in the CM2 domain that contribute to Cep215 self-assembly in cells. Mutations in these regions impair spindle assembly and the fidelity of chromosome segregation, thus highlighting the importance of Cep215 assembly. The requirement of multiple coiled-coil motifs and CM2-based interactions hint that worms, flies, and humans share a common mechanism for PCM scaffold assembly, despite using three very different proteins (SPD-5, Cnn, Cep215).

Comments for the author

I found many of the results to be interesting and useful to the centrosome community. However, the conclusions are too strong and over-interpreted. The main flaw of the paper is that the authors begin with a single model for assembly (e.g., Cep215 assembles through LLPS), then force all of their results into that model. No alternative models for assembly are presented or tested. Furthermore, the main experimental support of LLPS is fundamentally flawed: they see liquid-like behavior of Cep215 only when it is forced to assemble with the Cry2o light-inducible tag. Whether native Cep215 assembles through LLPS is never tested. They do perform FRAP on exogenously expressed mCherry-Cep215, which shows recovery, but this by itself is not a definitive test of the LLPS model.

Due to these problems, the manuscript is not acceptable for publication in any journal. However, there are so many good bits of data in this paper. With some re-writing and improved data analysis and presentation, this would be a good study.

I would consider a significantly revised manuscript that:
1. Focuses on identifying the interaction modules used by Cep215 for self-assembly, irrespective of the exact assembly mechanism. In the discussion, the authors could then make arguments for how their data fit into or challenge existing models for assembly (see PMID 31076235, 33248427). Fully testing the LLPS model would be too difficult and beyond the scope of this manuscript.
2. Includes larger images where the Cep215 foci are more easily visible. Many of the images are too small or dim, and I can't tell what is and what is not a Cep215 assembly.
3. Provides more in-depth, precise quantification of phenotypes. Throughout the manuscript, the authors report "% cells with condensates". Better metrics would include the number and brightness of foci per cells.

Other comments:
1. Introduction. Yeh et al. specifically downplay the role of phase separation and instead focused on the multivalent nature of Cep57. Please revise.
2. Figure 1A. Expression levels are higher in the transgenic line. Please state this in the text.
3. Figures 1C,D. A potential confounding factor is that PCM is growing (i.e., accumulating more proteins) during M phase. Can the authors distinguish true exchange of Cep215 from growth? One way is to test if the size of PCM increases over that time.
4. Figure 1C. This doesn't look like 50% recovery. How big is the ROI used for measurement?
5. Figure 2A. What does the top row represent? Is this a cell expressing mCherry-Cep215 without the cry2 tag? It doesn't say in the figure legend. Why are there so many bright blobs? Why do we see bright blobs, or even centrosome localization, in the Cep215-Cry2 expressing cell?
6. The use of the opto-droplet system to force accumulation of Cep215 is a misleading experiment. mCherry-Cry2 is known to assemble on its own; thus, it is not surprising that it will form droplets when fused to another sticky protein. I know that the authors performed at mCherry-Cry2 control (without fusing it Cep215) and this forms non-dynamic assemblies. However, this could be a concentration-dependent phenomenon; if levels are boosted, the mCherry-Cry2 may form liquid-like droplets (I've seen other labs show this, but I'm not sure if they published it).
7. I think the Cry2-tagging approach is still informative, especially for identifying self-assembly domains in Cep215. I advise that the authors tone down their conclusions. This approach cannot test if the native Cep215 undergoes LLPS.

© 2025. Published by The Company of Biologists under the terms of the Creative Commons Attribution License (https://creativecommons.org/licenses/by/4.0/).

8. Figure 4B. In the text, the authors point out that the condensates are "devoid" of gamma tubulin and PCNT. However, the data show that this is not the case. Rather, co-localization is diminished. Also, the authors should point out the fact that the F75A mutant clearly still binds gamma tubulin at the centrosome (white arrow).
9. Figure 5A. This figure should have a label on it stating "Alpha Fold model" so that readers don't think this is a solved structure. Also, the authors need to include the Predicted Aligned Error plot to demonstrate the confidence of the model.
10. Figure 5A. I question the validity of the AF3 model. The ITC experiments show that these two regions form a 1:1 complex, yet the AF3 model shows a tetramer of two dimers. Could the inclusion of the bulky MBP tags affect the stoichiometry of the complex? At the very least, the AF model should reflect the stoichiometry of the experimental result.
11. Figure 5C. The images are small, and it's hard to see the puncta.
12. Figure 6A. How were the coiled-coil regions determined? Did you use MARCOIL or another prediction? What were the parameters?
13. Figure 8. Why don't the authors simply measure Cep215 levels at the PCM? This would be a straightforward readout of how each domain contributes to PCM scaffold assembly.
14. Figure 2C. Is the dotted line the ROI? If so, it should be stated in the figure legend.
15. Figure 2C. is there a letter in the background behind the pictures?
16. I recommend making Figure 3 a supplemental figure.
17. Figure S3 should be a main figure.
18. Figure 5. How much does PCNT co-localize with Cep215?
19. Discussion. There is no evidence presented in this paper to conclude that native PCM is liquid-like. The authors have inferred liquid-like behavior based on a single FRAP experiment. FRAP can indicate dynamics (i.e., protein exchange), but that is separate from material properties (viscosity vs. elasticity). Keep in mind, that FRAP of tubulin in the spindle microtubules will reveal recovery because tubulin subunits are being added and lost; that is not proof of liquidity.
20. Discussion. "For instance, homotypic interactions of CCDs in CEP215 are known to exert autoinhibition against binding of the Î³-tubulin complex (Ohta et al., 2021; Tovey et al., 2021; Yang et al., 2023)." The first two studies are on SPD-5 and Cnn, not Cep215. Please be more specific.
21. Discussion. "In fact, PCNT itself also undergoes phase separation in a concentration-dependent manner (Jiang et al., 2021)." This study did not definitively prove that native, full-length PCNT could phase separate. They used truncations of PCNT in cells. Also, they never showed that full-length PCNT is sufficient to condense in vitro.
22. The authors demonstrate that multiple CCDs in Cep215 contribute to its self-assembly, supporting the model that multivalent coiled-coil interactions is a conserved assembly mechanism for the PCM scaffold. I recommend including this point in the discussion.
23. I personally would like the authors to emphasize that the F75A mutant does not affect gamma tubulin recruitment to PCM (Figure 8). There is an assumption in the centrosome field that this is a strong mutation that should block gamma tubulin binding, but it has never been tested in cells expressing full-length Cep215.

Reviewer 2

Advance summary and potential significance to field

In this interesting paper, the authors report that one of the PCM components, Cdk5rap2, has phase-separating properties. They also argue that this property is important for the formation of spindle poles during mitosis. The authors showed by FRAP experiments that Cep215 exhibits dynamic turn over during mitosis. Furthermore, by using an opto-droplet experimental system, they showed that Cep215 forms a dynamic liquid condensate. They also showed that the binding of Cep215 to PCNT via the CM2 domain of Cep215 is important for the formation of this condensate. This result was verified by experiments using PCNT knockout cells. Furthermore, the L1860S and L1867S mutants, in which mutations were introduced into amino acid residues important for the interaction between Cep215 and PCNTs, showed that the efficiency of condensate formation by Cep215 was greatly reduced. Furthermore, they generated various mutants lacking the coiled-coil region of Cep215 and found that several coiled-coil regions are important for condensate formation. Among these mutants, the CCD5 deletion and the L1867S mutants showed greatly reduced dynamics at the spindle poles during mitosis compared to the wild type. Furthermore, they observed the effects of various mutants on mitotic spindle formation under conditions in which they were expressed in cells to the same extent as endogenous Cep215. The results showed that mutants with reduced

© 2025. Published by The Company of Biologists under the terms of the Creative Commons Attribution License (https://creativecommons.org/licenses/by/4.0/).

efficiency of condensation formation of Cep215 formed a lower percentage of normal mitotic spindles. This trend was also observed in cells in which centriole was removed.
Together, the experiments in this paper detail the property of Cep215 to form a dynamic condensate when overexpressed in cells and the regions required for it to do so. On the other hand, the resolution of the analysis regarding the effect of this Cep215 property on the expansion and structure of the PCM and the formation of spindles during mitosis seems insufficient; it seems necessary to present a compelling data set regarding this point of physiological significance in order to warrant publication in the JCS. Specific points are listed below.

(major points)

1. The title claims that Cep215 phase separation is essential for spindle formation, but this is misleading. The criteria for determining the abnormality of spindle formation that are shown in Figure 8 are not clear (e.g., Bipole-mis). In the case of neither monopolar nor multipolar, it should be clarified by what index the spindle formation is considered to be abnormal. In addition, the phenotypic examples of CCDC2, CCDC5, and L1867S mutants (Figure 8A) show examples of multipolar spindles, but from the quantitative data, this population should not be so common in each. So, it is not an appropriate example of a representative phenotype. Also, in these examples, the mutant proteins may or may not localize to the spindle poles, but can this be reasonably explained?
2. Also, if a model like the one in Figure 10 is to be described, a detailed analysis of the spindle poles and PCM when various mutants of Cep215 are expressed would be necessary. The data in Figures 8 and 9 are not of sufficient resolution to support that the phase-separating nature of Cep215 is indeed necessary for functional spindle poles and spindle formation.
3. In Figure 1, GFP-Cep215 dynamics are higher in mitosis, but is this also PCNT-dependent? Furthermore, in the CCD5 deletion mutant, there is little or no dynamics of Cep215 at spindle poles in mitosis. The phenotype of mitotic spindle formation is also most pronounced in the CCD5 deletion mutant. What are the mechanisms by which the CCD5 region is involved in the efficiency of condensation of Cep215 and in the structure of the PCM and in its ability to form microtubules? These details should be experimentally addressed.

(minor points)

4. In Figure 2-3, Cep215 seems to be able to induce condensate formation without the CRY2 tag, but are these condensates characterized as fusing droplets?
5. In Figure 4C, do Cep215 condensates without CRY2 tags show similar PCNT and gamma-Tub localization patterns?
6. In Figure 6, what were the aims of CCD1-5 selected to target?

Reviewer 3

Advance summary and potential significance to field

In this study, the authors try to determine whether the key centrosomal protein CEP215 (also known as CDK5RAP2) undergoes phase separation, how this potential phase separation is mediated, and whether it is important during centrosome assembly. Previous studies have shown that the C. elegans homologue, SPD-5, undergoes phase separation to help regulate centrosome assembly, and this current study appears to test the hypothesis that a similar process occurs in human cells, and that this process is facilitated by an interaction with Pericentrin. To test if CEP215 phase separates, they tag it with a light-inducible oligomerisation domain (CRY2) and use light to stimulate the formation of cytosolic clusters (which the authors refer to as condensates). They then test whether these clusters form under different conditions, such as when certain binding domains are mutated or when another key centrosomal protein, PCNT, is absent. Having found mutants of CEP215 that disrupt cytosolic clustering, they examine the ability of these mutants to enable centrosome assembly and spindle formation, concluding that phase separation of CEP215 is important for these processes. While the paper addresses an interesting question, and has nice data on how CEP215 and Pericentrin interact, there are key issues that need addressing prior to publication.

Comments for the author

© 2025. Published by The Company of Biologists under the terms of the Creative Commons Attribution License (https://creativecommons.org/licenses/by/4.0/).

Major concerns
1) The authors do not test whether CEP215 can phase separate independently of the CRY2 domain. In the OptoDroplets paper that the authors base their assay on, Shin et al. tag with CRY2 the small IDR regions from RNP body proteins that are already known to phase separate, using the CRY2 tag as a means to spatiotemporally control phase separation in cells with light. In this current paper, the authors are tagging full-length CEP215 with CRY2 to drive cytosolic clustering, without first demonstrating that CEP215 has an intrinsic propensity to phase separate. Could they not do this by purifying the protein and performing phase separation tests in vitro?
2) The authors should also do a key control that I think was lacking from the Shin et al., 2017 paper to prove the OptoDroplets assay is specific to proteins that phase separate. They should tag a protein not believed to phase separate with CRY2 and show that this does not induce cytosolic clusters after light activation.
3) Once they have established CEP215 can phase separate, the authors should re-test their mutations that they believe interfere with phase separation (in the new phase separation assays with untagged CEP215).
4) The structural prediction of the CEP215 and Pericentrin interaction domains looks remarkably similar to the PReM-CM2 interaction for fly Cnn reported in Feng et al., 2017, Cell. Can the authors comment on this? Is this region of CEP215 the PReM domain? Does it also interact with the CM2 domain of CEP215? Could Pericentrin and CM2 be competing for binding to this region?
5) When the authors use "phase separation mutations" to conclude that phase separation of CEP215 is important for centrosome and mitotic spindle assembly, they need to consider the possibility that they are mutating regions of Cep215 that interact with other PCM proteins - this means the failure to properly assemble centrosomes does not necessarily have anything to do with phase separation, as one cannot distinguish whether it is the ability of Cep215 to phase separate, or the ability of it to form important connections with other major PCM components.

Minor concerns
6) "Phase separation mechanisms have been implicated in assembly of centrosomes, non-membranous organelles (Jiang et al., 2021; Yeh et al., 2024)" - I think the Woodruff papers should be cited here as they were the first to show phase separation of a centrosome protein.
7) The authors say that "the expression levels of ectopic FLAG-mCh-CEP215 were comparable to those of endogenous CEP215 in control cells (Fig. 1A)", but the ectopic CEP215 appears to be more strongly expressed - have the authors quantified this?
8) The authors say that "Similar to endogenous CEP215, FLAG-mCh-CEP215 was localized to the centrosomes, and the level of accumulation at the centrosome changed during the cell cycle, such that it was minimal at S phase, started to increase at G2 phase, and reached the highest level at M phase (Fig. 1B)." It seems to me that the highest level in both cases is in G2, not M phase. Have the authors quantified this?
9) "Consistent with these, we found that cytoplasmic condensates of FLAG-CEP215F75A-CRY2 and FLAG-CEP215Î”CM2-CRY2 are devoid of Î³-tubulin and PCNT signals, respectively (Fig. 4B, C)." From the picture, it seems as though g-tub is localising to the puncta in F75A mutants.
10) "Interestingly, as we examined the light-induced condensation of CEP215 variants, we noticed that cytoplasmic condensation was highly reduced in the construct of CM2 deletion, FLAG-CEP215Î”CM2-CRY2, suggesting that CM2 is critical for the phase separation of CEP215 in cells (Fig. 4D; Fig. S2A). This result also implies that the association of PCNT, rather than the CM2-mediated self-association per se, may play an important role in the phase separation of CEP215." I'm not sure why this result would suggest a role for Pericentrin and not CM2 oligomerisation?
11) "When the binding domains of CEP215 (MBP-CEP215 1814-1877) and PCNT (MBP- PCNT 2388-2420) were bacterially expressed, purified and mixed in vitro, they instantly form a complex (Fig. S3A)." These protein fragments are similar in size and so could be expected to elute from the SEC column at a similar volume even if not in a complex.
12) "These results suggest that the intrinsic phase separation ability of CEP215 relies on intact CCD structures". So far, the authors have not shown that phase separation ability is intrinsic, as they have performed the experiments with a CRY2 domain.

First revision

© 2025. Published by The Company of Biologists under the terms of the Creative Commons Attribution License (https://creativecommons.org/licenses/by/4.0/).

Author response to reviewers' comments

Reviewer 1: SUMMARY
OF THE ADVANCE MADE IN THIS PAPER AND ITS POTENTIAL SIGNIFICANCE TO THE FIELD
Yoo et al. seek to reveal how the human centrosome assembles at the molecular level. They focus on assembly of the pericentriolar material (PCM), which comprises the bulk of the centrosome and serves as a scaffold to recruit microtubule nucleating and anchoring factors. Much of the mechanistic insight into PCM assembly has been determined through biochemical studies in worms and flies, and it is not clear if those principles apply in distantly related mammals. Thus, the authors are addressing an important topic.
The authors claim that PCM assembles through liquid-liquid phase separation of Cep215. They further identify several domains (two coiled-coil regions) and select amino acids in the CM2 domain that contribute to Cep215 self-assembly in cells. Mutations in these regions impair spindle assembly and the fidelity of chromosome segregation, thus highlighting the importance of Cep215 assembly. The requirement of multiple coiled-coil motifs and CM2-based interactions hint that worms, flies, and humans share a common mechanism for PCM scaffold assembly, despite using three very different proteins (SPD-5, Cnn, Cep215).
SUGGESTIONS TO AUTHORS
I found many of the results to be interesting and useful to the centrosome community. However, the conclusions are too strong and over-interpreted. The main flaw of the paper is that the authors begin with a single model for assembly (e.g., Cep215 assembles through LLPS), then force all of their results into that model. No alternative models for assembly are presented or tested. Furthermore, the main experimental support of LLPS is fundamentally flawed: they see liquid-like behavior of Cep215 only when it is forced to assemble with the Cry2 light-inducible tag. Whether native Cep215 assembles through LLPS is never tested. They do perform FRAP on exogenously expressed mCherry-Cep215, which shows recovery, but this by itself is not a definitive test of the LLPS model.
Due to these problems, the manuscript is not acceptable for publication in any journal. However, there are so many good bits of data in this paper. With some re-writing and improved data analysis and presentation, this would be a good study.
I would consider a significantly revised manuscript that:
We appreciate for all the constructive comments. We admit that some of the results are overinterpreted. Especially, LLPS should not be concluded with the FRAP analyses only. In the revised manuscript, we include additional experiments which were suggested by the reviewers, and extensively re-wrote the text. The follows are responses to the specific points.

1. Focuses on identifying the interaction modules used by Cep215 for self-assembly, irrespective of the exact assembly mechanism. In the discussion, the authors could then make arguments for how their data fit into or challenge existing models for assembly (see PMID 31076235, 33248427). Fully testing the LLPS model would be too difficult and beyond the scope of this manuscript.

A number of previous reports indicate that CEP215 forms homomultimers (Yang et al., JCB, 222:e202007101, 2023). In this manuscript, we specifically focused on the LZ:CM2 homotypic interaction which had been confirmed in *Drosophila* Cnn (Feng et al., 2017). We performed coimmunoprecipitation assays with CEP215$^{611-670}$ and CEP215$^{1661-1893}$ which potentially correspond to the LZ and CM2 domains of human CEP215, respectively (Feng et al., 2017). First, we observed that both CEP215$^{611-670}$ and CEP215$^{1661-1893}$ formed homo-multimers, suggesting they participate in homophilic interactions of CEP215 (Fig. 5E). However, HA- CEP215$^{611-670}$ was not coimmunoprecipitated with FLAG-CEP215$^{1661-1893}$, nor was HA- CEP215$^{1661-1893}$ with FLAG-CEP215$^{611-670}$ (Fig. 5E). These results suggest that the human CEP215 may not form the LZ:CM2 tetramer which have been proposed in *Drosophila* Cnn (Feng et al., 2017). Even if we did not find experimental evidence supporting the LZ:CM2 interaction, we are sure that CEP215 can form homo-polymers in a sequence-specific manner.

2. Includes larger images where the Cep215 foci are more easily visible. Many of the images are too small or dim, and I can't tell what is and what is not a Cep215 assembly.

We apologize the unreadable images in the manuscript. As suggested, we changed many images which are more visible. For example, we adopted better images of Figs. 3B and 5C in the revised manuscript. For Fig. 9A, we showed representative spindles of monopole, intact bipole, bipole with lagging chromosomes, bipole with mis-segregated chromosomes, and multipole spindles.

© 2025. Published by The Company of Biologists under the terms of the Creative Commons Attribution License (https://creativecommons.org/licenses/by/4.0/).

Representative centrosome signals of the PCM proteins were also presented in Fig. S9.

3. Provides more in-depth, precise quantification of phenotypes. Throughout the manuscript, the authors report "% cells with condensates". Better metrics would include the number and brightness of foci per cells.
We initially tried to quantify the sizes and intensities of the cytoplasmic condensates of FLAG-CEP215-CRY2, but realized that it is almost impossible. Instead, we noticed that the cytoplasmic condensates are formed, dependent on the amount of the ectopic proteins and duration of light activation. Therefore, we decided to quantify the time-course changes in number of cytoplasmic condensate-positive cells at proper amounts of the ectopic proteins. Representative cytoplasmic condensates and time-course quantifications are now shown in Figs. S1 and S2.

Other comments:
1. Introduction. Yeh et al. specifically downplay the role of phase separation and instead focused on the multivalent nature of Cep57. Please revise.
We appreciate for the thoughtful comment. As suggested, we carefully referred Yeh et al. paper in the revised manuscript.

2. Figure 1A. Expression levels are higher in the transgenic line. Please state this in the text.
As suggested, we quantified intensities of the CEP215-specific band in the immunoblots presented in Fig. 1A. The results showed that the band intensities of FLAG-CEP215 is stronger by 1.7 times than the endogenous CEP215. This point is addressed in the results section of the revised manuscript.

3. Figures 1C,D. A potential confounding factor is that PCM is growing (i.e., accumulating more proteins) during M phase. Can the authors distinguish true exchange of Cep215 from growth? One way is to test if the size of PCM increases over that time.
As suggested, we carefully analyzed the FRAP recovery of mCh-CEP215 at mitotic spindle poles. As shown in Fig. 1E, the signaling area of mCh-CEP215 was quickly recovered, such that about a half of the original area was recovered within 120 sec after the bleaching (Fig. 1E). On the other hand, the signaling intensity of mCh-CEP215 was recovered much slower than the signaling area during the same time period (Fig. 1E). Similar results were observed in three repeated experiments (data not shown). These results suggest that mCh-CEP215 may be exchanged, irrespective of the relative positions within the spindle poles.

4. Figure 1C. This doesn't look like 50% recovery. How big is the ROI used for measurement?
The ROI in Fig. 1D is about 25μm^2 circle. We generated Fig. 1D with repeated the experiments followed by statistical analyses.

5. Figure 2A. What does the top row represent? Is this a cell expressing mCherry-Cep215 without the cry2 tag? It doesn't say in the figure legend. Why are there so many bright blobs? Why do we see bright blobs, or even centrosome localization, in the Cep215-Cry2 expressing cell?
As pointed out, the top row of Fig. 2A represents live fluorescent images of mCh-CEP215 which are ectopically expressed in HeLa cells. The blobs in the top row are believed to be aggregates, which are different from cytoplasmic condensates in the second row in terms that
(1) they are already formed before the light activation, (2) they are diverse in shapes and sizes, and (3) they do not coalesce among themselves but stay unchanged.
As suggested, we improved labeling of the figures and explained the observations in the results section of the revised manuscript.

6. The use of the opto-droplet system to force accumulation of Cep215 is a misleading experiment. mCherry-Cry2 is known to assemble on its own; thus, it is not surprising that it will form droplets when fused to another sticky protein. I know that the authors performed at mCherry-Cry2 control (without fusing it Cep215) and this forms non-dynamic assemblies. However, this could be a concentration-dependent phenomenon; if levels are boosted, the mCherry-Cry2 may form liquid-like droplets (I've seen other labs show this, but I'm not sure if they published it).
Yes, we also observed that mCh-CRY2 forms liquid-like droplets in time and concentration-dependent manners. However, in our experimental conditions in Fig. 2C, mCh-CEP215-CRY2 formed opto-droplets whereas mCh-CRY2 did not, supporting the hypothesis that CEP215 has

© 2025. Published by The Company of Biologists under the terms of the Creative Commons Attribution License (https://creativecommons.org/licenses/by/4.0/).

intrinsic property to undergo phase separation. We would like to emphasize that the mCh-CRY2 system was primarily used to screen phase-separation-defect candidates among the CEP215 mutants.

7. I think the Cry2-tagging approach is still informative, especially for identifying self- assembly domains in Cep215. I advise that the authors tone down their conclusions. This approach cannot test if the native Cep215 undergoes LLPS.

In this manuscript, we adopted the CRY2-tagging approach to have clues in molecular dynamics of CEP215 and to screen mutants with defects in FRAP recovery. As suggested, we carefully described the significance of the CRY2-tagging approach in the results section of the revised manuscript.

8. Figure 4B. In the text, the authors point out that the condensates are "devoid" of gamma tubulin and PCNT. However, the data show that this is not the case. Rather, co-localization is diminished. Also, the authors should point out the fact that the F75A mutant clearly still binds gamma tubulin at the centrosome (white arrow).

First of all, we changed to better representing images of Fig. 3B, so that one can clearly distinguish specific signals of FLAG, γ-tubulin and PCNT. As pointed out, co-localization of γ-tubulin and PCNT are diminished in cytoplasmic condensates of CEP215-CRY2^{F75A} and CEP215-CRY2$^{\Delta CM2}$, respectively (Fig. 3B). It is interesting that γ-tubulin is detected at the centrosomes in CEP215-CRY2^{F75A}-substituted cells, probably through other centrosome proteins, such as CEP192. We also observed microtubule re-grew from the condensates (Fig. S3).

9. Figure 5A. This figure should have a label on it stating "Alpha Fold model" so that readers don't think this is a solved structure. Also, the authors need to include the Predicted Aligned Error plot to demonstrate the confidence of the model.

As you pointed out, we correct the figure including the labels to remove further readers' confusion. As pointed out, we included the Expected Position Error plot to demonstrate confidence of the model (Fig. S4).

10. Figure 5A. I question the validity of the AF3 model. The ITC experiments show that these two regions form a 1:1 complex, yet the AF3 model shows a tetramer of two dimers. Could the inclusion of the bulky MBP tags affect the stoichiometry of the complex? At the very least, the AF model should reflect the stoichiometry of the experimental result.

We used the MBP tags to increase the solubility of the CEP215 CM2 domain. The SEC-MALS analysis revealed that both MBP-CEP215$^{1661\text{-}1893}$ and PCNT$^{2350\text{-}2450}$ formed dimers (Fig. 4D). Based on these findings along with the ITC data, we predicted that these two proteins interact in a 2:2 ratio as shown in Fig. 5A. Homo-multimer formation ability of CEP215$^{1661\text{-}1893}$ was also observed in the coimmunoprecipitation assays in Fig. 5E.

11. Figure 5C. The images are small, and it's hard to see the puncta.

We apologize for the unreadable figures. As pointed out, we changed to better representing images of Fig. 5C in the revised manuscript.

12. Figure 6A. How were the coiled-coil regions determined? Did you use MARCOIL or another prediction? What were the parameters?

We used PCOILS and PONDR to analyze the coiled-coil regions and the intrinsically disordered region of CEP215, respectively. The MTIDK matrix was used as a PCOILS parameter.

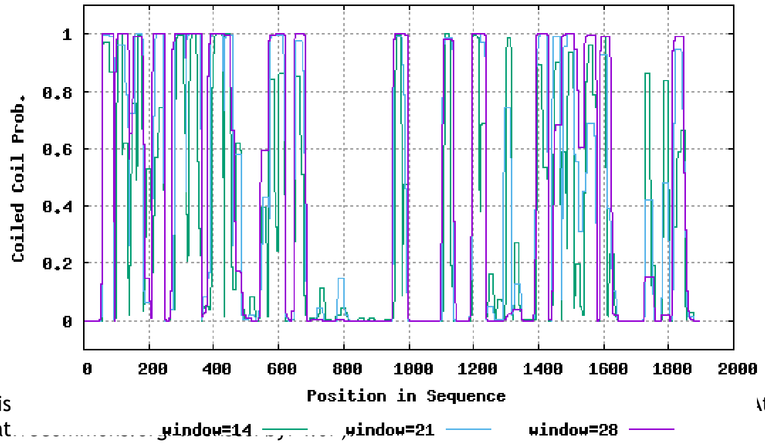

© 2025. Publis[illegible] Attribution License (https://creat[illegible]

PCOILS

PONDR

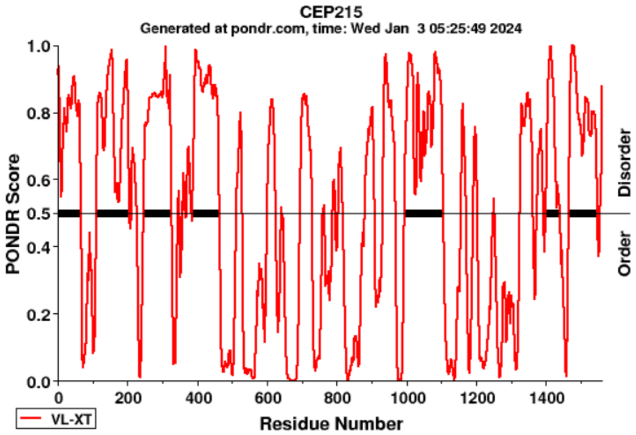

13. Figure 8. Why don't the authors simply measure Cep215 levels at the PCM? This would be a straightforward readout of how each domain contributes to PCM scaffold assembly.

As pointed out, we determined spindle pole intensities of CEP215 and PCNT in HeLa cells whose endogenous CEP215 was substituted with ectopic mCh-FLAG-CEP215 mutants. The results showed that the spindle pole levels of FLAG-mCh-CEP215$^{\square CCD5}$ and FLAG-mCh- CEP215^{L1867S} were significantly reduced, suggesting that molecular dynamics of CEP215 need to be up-regulated for centrosome accumulation in mitotic cells (Fig. 8A, B). Furthermore, spindle pole levels of PCNT were also reduced in *CEP215* KO cells and FLAG-mCh- CEP215^{L1867S}-expressing cells, suggesting that CEP215 is partially involved in PCNT recruitment in mitotic spindle pole formation (Fig. 8A, C). We previously reported that the centrosome sizes are larger in late G2 phase than those in mitosis (Lee and Rhee, 2010). We interpret the results that selective PCM proteins are recruited to the centrosomes at G2 phase, but need to be reorganized for functional spindle poles. We propose that multimeric interactions of CEP215 may be a part of the reorganization process of spindle poles. To test the hypothesis, we determined the sizes of centrosomes in HeLa cells with CEP215 mutant proteins during the cell cycle. The results showed that the centrosome areas of CEP215 and PCNT was enlarged at G2 phase and eventually reduced in M phase of the control HeLa cells, whereas those of γ-tubulin and CEP192 steadily increased as reported previously (Figs. 8D and S8). However, the centrosome sizes of the FLAG-mCh-CEP215$^{\square CCD5}$ and FLAG-mCh-CEP215^{L1867S} expressing cells were generally reduced in the G2 phase and M phase cells (Fig. 8D). These results suggest that multimeric interactions of CEP215 is important for centrosome recruitment of PCM proteins during mitosis.

14. Figure 2C. Is the dotted line the ROI? If so, it should be stated in the figure legend.
As predicted, the dotted circles indicate region of irradiance where the photo bleached. As suggested, we explained the meaning of the dotted circles in the legend of Fig. 2 in the revised manuscript.

15. Figure 2C. is there a letter in the background behind the pictures?
We are shamed of our sloppiness. The letter is now removed.

16. I recommend making Figure 3 a supplemental figure.
As suggested, detection of cytoplasmic condensates of FLAG-mCh-CEP215-CRY2 is shown in Figs. S1 and S2 of the revised manuscript.

17. Figure S3 should be a main figure.
As suggested, the specific interaction of PCNT and CEP215 fragment is shown in Fig. 5 of the revised manuscript.

18. Figure 5. How much does PCNT co-localize with Cep215?

© 2025. Published by The Company of Biologists under the terms of the Creative Commons Attribution License (https://creativecommons.org/licenses/by/4.0/).

Comparing input vs coimmunoprecipitated PCNT, about one-tenth of endogenous PCNT is associated with ectopic FLAG-CEP215.

19. Discussion. There is no evidence presented in this paper to conclude that native PCM is liquid-like. The authors have inferred liquid-like behavior based on a single FRAP experiment. FRAP can indicate dynamics (i.e., protein exchange), but that is separate from material properties (viscosity vs. elasticity). Keep in mind, that FRAP of tubulin in the spindle microtubules will reveal recovery because tubulin subunits are being added and lost; that is not proof of liquidity.
We agree that additional experimental supports are required for us to conclude the liquid-like state of CEP215 at the centrosomes. Therefore, we described that diffusive dynamics of CEP215 is upregulated in mitotic spindle poles. We also toned down our conclusions.

20. Discussion. "For instance, homotypic interactions of CCDs in CEP215 are known to exert autoinhibition against binding of the γ-tubulin complex (Ohta et al., 2021; Tovey et al., 2021; Yang et al., 2023)." The first two studies are on SPD-5 and Cnn, not Cep215. Please be more specific.
As suggested, we carefully referred the papers in the revised manuscript.

21. Discussion. "In fact, PCNT itself also undergoes phase separation in a concentration-dependent manner (Jiang et al., 2021)." This study did not definitively prove that native, full-length PCNT could phase separate. They used truncations of PCNT in cells. Also, they never showed that full-length PCNT is sufficient to condense in vitro.
As suggested, we carefully referred Jiang et al. (2021) in the revised manuscript.

22. The authors demonstrate that multiple CCDs in Cep215 contribute to its self-assembly, supporting the model that multivalent coiled-coil interactions is a conserved assembly mechanism for the PCM scaffold. I recommend including this point in the discussion.
As suggested, we addressed this point in the Discussion section of the revised manuscript as follow: Our results with multiple CEP215 mutants with disrupted CCDs suggest that multivalent coiled-coil interactions is important for assembly and maintenance of mitotic spindle poles.

23. I personally would like the authors to emphasize that the F75A mutant does not affect gamma tubulin recruitment to PCM (Figure 8). There is an assumption in the centrosome field that this is a strong mutation that should block gamma tubulin binding, but it has never been tested in cells expressing full-length Cep215.
We observed that γ-tubulin was not recruited to the cytoplasmic condensates of the mCh-CEP215^{F75A}-CRY2, revealing importance of phenylalanine residue at 75th position of CEP215 with γ-TuRC (Fig. 4C). However, FLAG-mCh-CEP215^{F75A}-substituted cells have normal mitotic spindles, suggesting that specific interactions of CEP215 with γ-TuRC may be dispensable for centrosome recruitment of γ-tubulin into the centrosomes and mitosis (Fig. 9).

Reviewer 2: In this interesting paper, the authors report that one of the PCM components, Cdk5rap2, has phase-separating properties. They also argue that this property is important for the formation of spindle poles during mitosis. The authors showed by FRAP experiments that Cep215 exhibits dynamic turn over during mitosis. Furthermore, by using an opto-droplet experimental system, they showed that Cep215 forms a dynamic liquid condensate. They also showed that the binding of Cep215 to PCNT via the CM2 domain of Cep215 is important for the formation of this condensate. This result was verified by experiments using PCNT knockout cells. Furthermore, the L1860S and L1867S mutants, in which mutations were introduced into amino acid residues important for the interaction between Cep215 and PCNTs, showed that the efficiency of condensate formation by Cep215 was greatly reduced. Furthermore, they generated various mutants lacking the coiled-coil region of Cep215 and found that several coiled-coil regions are important for condensate formation. Among these mutants, the CCD5 deletion and the L1867S mutants showed greatly reduced dynamics at the spindle poles during mitosis compared to the wild type. Furthermore, they observed the effects of various mutants on mitotic spindle formation under conditions in which they were expressed in cells to the same extent as endogenous Cep215. The results showed that mutants with reduced efficiency of condensation formation of Cep215 formed a lower percentage of normal mitotic spindles. This trend was also observed in cells in which centriole was removed.

© 2025. Published by The Company of Biologists under the terms of the Creative Commons Attribution License (https://creativecommons.org/licenses/by/4.0/).

Together, the experiments in this paper detail the property of Cep215 to form a dynamic condensate when overexpressed in cells and the regions required for it to do so. On the other hand, the resolution of the analysis regarding the effect of this Cep215 property on the expansion and structure of the PCM and the formation of spindles during mitosis seems insufficient; it seems necessary to present a compelling data set regarding this point of physiological significance in order to warrant publication in the JCS. Specific points are listed below.

We are grateful of the critical comments. We include additional data which explain importance of multivalent interactions of CEP215 in mitotic spindle pole assembly and functions. Detailed results are described in response to specific critics as below.

(major points)

1. The title claims that Cep215 phase separation is essential for spindle formation, but this is misleading. The criteria for determining the abnormality of spindle formation that are shown in Figure 8 are not clear (e.g., Bipole-mis). In the case of neither monopolar nor multipolar, it should be clarified by what index the spindle formation is considered to be abnormal. In addition, the phenotypic examples of CCDC2, CCDC5, and L1867S mutants (Figure 8A) show examples of multipolar spindles, but from the quantitative data, this population should not be so common in each. So, it is not an appropriate example of a representative phenotype. Also, in these examples, the mutant proteins may or may not localize to the spindle poles, but can this be reasonably explained?

Abnormalities of spindles have been adapted as described by Yang et al. (J. Neurosci 23:10454, 2003). In brief, we defined bipoles with mis-segregated and lagging chromosomes when chromosomes are detected outside or inside the spindles, respectively. In the revised manuscript, we explained spindle defects with representative images (Fig. 9A).

The spindle defect phenotypes in the CCDC2, CCDC5, and L1867S mutants may be mild but consistently significant in mitotic cells prepared with three different ways, such as drug-treatment (Fig. 9B), natural (Fig. 9C) and centriole-less conditions (Fig. 9D). These results suggest that dynamic molecular interactions of CEP215 may affect proper spindle formation to some extent.

In addition, we determined spindle pole localization of the CEP215 mutants. The results showed that the spindle pole levels of FLAG-mCh-CEP215$^{\square CCD5}$ and FLAG-mCh- CEP215^{L1867S} were significantly reduced, suggesting that molecular dynamics of CEP215 need to be changed for centrosome accumulation in mitotic cells (Fig. 8A, B). Furthermore, spindle pole levels of PCNT was also reduced in *CEP215* KO cells and FLAG-mCh-CEP215^{L1867S}- expressing cells, suggesting that CEP215 is partially involved in PCNT recruitment in mitotic spindle pole formation (Fig. 8A, C).

2. Also, if a model like the one in Figure 10 is to be described, a detailed analysis of the spindle poles and PCM when various mutants of Cep215 are expressed would be necessary. The data in Figures 8 and 9 are not of sufficient resolution to support that the phase-separating nature of Cep215 is indeed necessary for functional spindle poles and spindle formation.

We previously reported that the centrosome sizes are larger in late G2 phase than those in mitosis (Lee and Rhee, 2010). We interpret the results that selective PCM proteins are recruited to the centrosomes at G2 phase, but need to be reorganized for functional spindle poles. We propose that multivalent interactions of CEP215 may be involved in spindle pole formation and maintenance. To test the hypothesis, we determined sizes of the PCM proteins in HeLa cells whose endogenous CEP215 is substituted with the FLAG-mCh-CEP215 mutant proteins. The results showed that the centrosome areas of CEP215 and PCNT was enlarged at G2 phase and eventually reduced in M phase of the control HeLa cells, whereas those of γ-tubulin and CEP192 steadily increased as reported previously (Figs. 8D and S8). However, the centrosome sizes of the FLAG-mCh-CEP215$^{\square CCD5}$- and FLAG-mCh-CEP215^{L1867S}-expressing cells were generally reduced in the G2 phase and M phase cells (Fig. 8D). These results suggest that multivalent interactions of CEP215 is important for centrosome recruitment of PCM proteins as well as CEP215 itself during mitosis.

3. In Figure 1, GFP-Cep215 dynamics are higher in mitosis, but is this also PCNT-dependent? Furthermore, in the CCD5 deletion mutant, there is little or no dynamics of Cep215 at spindle poles in mitosis. The phenotype of mitotic spindle formation is also most pronounced in the

© 2025. Published by The Company of Biologists under the terms of the Creative Commons Attribution License (https://creativecommons.org/licenses/by/4.0/).

CCD5 deletion mutant. What are the mechanisms by which the CCD5 region is involved in the efficiency of condensation of Cep215 and in the structure of the PCM and in its ability to form microtubules? These details should be experimentally addressed.

Since the fluorescent recovery rates of both FLAG-mCh-CEP215$^{\square CCD5}$- and FLAG-mCh-CEP215^{L1867S}-substituted cells were reduced, we propose that both multivalent interactions of CEP215 CCDs and specific interaction of CEP215 with PCNT are important for CEP215 dynamicity in mitotic spindles (Fig. 7B, C). FLAG-mCh-CEP215$^{\square CCD5}$ was not efficiently recruited to the mitotic centrosomes (Fig. 8A, B). We also observed that other PCM proteins were not efficiently recruited to the centrosomes in FLAG-mCh-CEP215$^{\square CCD5}$-substituted cells (Fig. 8D). These results suggest that impairment of molecular dynamics of CEP215 results in reduction of centrosome accumulation of the protein for mitotic spindle poles. We explained this point in the Discussion section of the revised manuscript.

(minor points)

4. In Figure 2-3, Cep215 seems to be able to induce condensate formation without the CRY2 tag, but are these condensates characterized as fusing droplets?

We can distinguish liquid like condensates from solid aggregates based on the fact that (1) they are already formed before the light activation, (2) they are diverse in shapes and sizes, and (3) they do not coalesce among themselves but stay unchanged. As shown in Fig. 2A, the signals in the mCh-CEP group are mostly from solid aggregates.

5. In Figure 4C, do Cep215 condensates without CRY2 tags show similar PCNT and gamma- Tub localization patterns?

We can distinguish liquid-like condensates from solid aggregates in the immunostaining of the ectopic FLAG-mCh-CEP215 in the cytoplasm (Fig. S2A). The FLAG-CEP215 aggregates are devoid of other PCM proteins such as PCNT and γ-tubulin (Attached Fig. A1). This point is addressed in the results section of the revised manuscript.

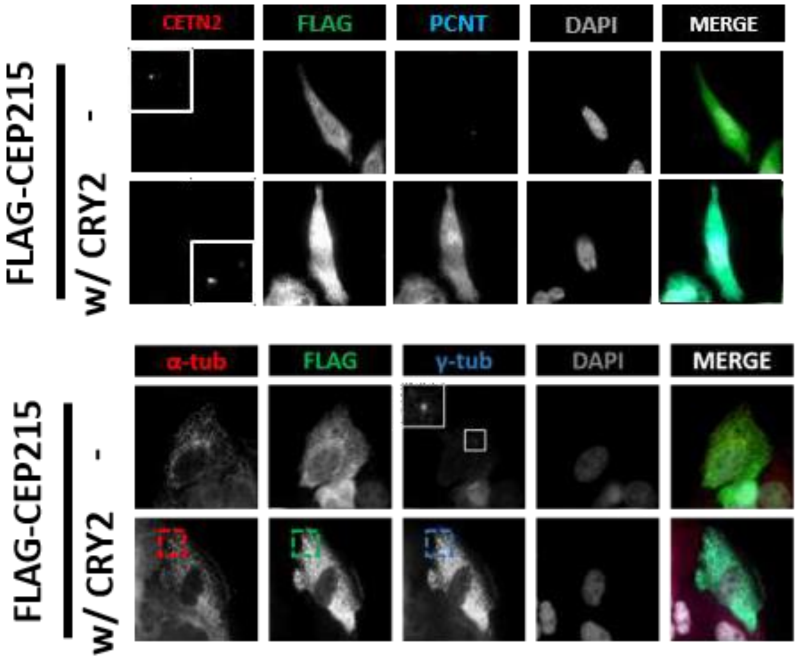

Attached Figure A1. Immunostaining analyses of FLAG-CEP215 with and without CRY2 FLAG-CEP215 and FLAG-CEP215-CRY2 were transiently expressed in HeLa cells and immunostained with FLAG, PCNT, γ-tubulin, centrin-2, centrin-2, and α-tubulin antibodies. DNA was stained with DAPI.

6. In Figure 6, what were the aims of CCD1-5 selected to target?

We wished to isolate truncated mutants which have defects in an intrinsic phase separation property of CEP215. Among many candidates whose deletions might result in disruption of CCD

© 2025. Published by The Company of Biologists under the terms of the Creative Commons Attribution License (https://creativecommons.org/licenses/by/4.0/).

interactions with other PCM proteins, we chose ¤CCD1-5 which best fit for our criteria.

Reviewer 3: SUMMARY OF THE ADVANCE MADE IN THIS PAPER AND ITS POTENTIAL SIGNIFICANCE TO THE FIELD

In this study, the authors try to determine whether the key centrosomal protein CEP215 (also known as CDK5RAP2) undergoes phase separation, how this potential phase separation is mediated, and whether it is important during centrosome assembly. Previous studies have shown that the C. elegans homologue, SPD-5, undergoes phase separation to help regulate centrosome assembly, and this current study appears to test the hypothesis that a similar process occurs in human cells, and that this process is facilitated by an interaction with Pericentrin. To test if CEP215 phase separates, they tag it with a light-inducible oligomerisation domain (CRY2) and use light to stimulate the formation of cytosolic clusters (which the authors refer to as condensates). They then test whether these clusters form under different conditions, such as when certain binding domains are mutated or when another key centrosomal protein, PCNT, is absent. Having found mutants of CEP215 that disrupt cytosolic clustering, they examine the ability of these mutants to enable centrosome assembly and spindle formation, concluding that phase separation of CEP215 is important for these processes. While the paper addresses an interesting question, and has nice data on how CEP215 and Pericentrin interact, there are key issues that need addressing prior to publication.

SUGGESTIONS TO AUTHORS

Major concerns

1) The authors do not test whether CEP215 can phase separate independently of the CRY2 domain. In the OptoDroplets paper that the authors base their assay on, Shin et al. tag with CRY2 the small IDR regions from RNP body proteins that are already known to phase separate, using the CRY2 tag as a means to spatiotemporally control phase separation in cells with light. In this current paper, the authors are tagging full-length CEP215 with CRY2 to drive cytosolic clustering, without first demonstrating that CEP215 has an intrinsic propensity to phase separate. Could they not do this by purifying the protein and performing phase separation tests in vitro?

We wished to investigate intrinsic ability of CEP215 to undergo phase separation in vitro with purified proteins. However, it was not possible to purify biologically active CEP215 proteins in our hand, due to its size reaching to 215 kDa. Therefore, we only investigated molecular dynamics of CEP215 in cells with FRAP analysis. When a comparable amount of ectopic CEP215 proteins are expressed in a cell, majority of them are concentrated at the centrosome. FRAP analyses revealed that CEP215 form solid lattice in interphase centrosomes, but are movable in mitotic centrosomes (Figure 1). These observations suggest that CEP215 may be more dynamic in the mitotic centrosomes. Since we were not able to perform in vitro experiments for phase separation property of CEP215, we changed the title and toned down our conclusion in the revised manuscript.

2) The authors should also do a key control that I think was lacking from the Shin et al., 2017 paper to prove the OptoDroplets assay is specific to proteins that phase separate. They should tag a protein not believed to phase separate with CRY2 and show that this does not induce cytosolic clusters after light activation.

We understand that the CRY2 domain itself can form opto-droplets at appropriate conditions. However, we found that the CEP215-CRY2 protein behaves like liquid with a strong FRAP recovery pattern, even if CRY2 forms aggregates at the same condition (Fig. 2C, D). These results suggest that CEP215 has an intrinsic ability to undergo phase separation. Nonetheless, we accept that additional experiments are required to confirm the phase separation property of CEP215, including a series of in vitro tests with purified proteins.

3) Once they have established CEP215 can phase separate, the authors should re-test their mutations that they believe interfere with phase separation (in the new phase separation assays with untagged CEP215).

It is unfortunate that we cannot purify biologically active CEP215 proteins to be used for phase separation experiments in vitro. Instead, we performed FRAP analyses at the centrosomes of the cells whose endogenous CEP215 is substituted with mCh-FLAG-CEP215 mutants. The results revealed that molecular dynamics of CEP215 is upregulated at the centrosome of mitotic cells (Fig. 1). Furthermore, we found that dynamics of CEP215 is regulated by low affinity interactions (through CCDs) as well as a high affinity interaction (with PCNT) in the mitotic spindle poles (Fig. 7).

© 2025. Published by The Company of Biologists under the terms of the Creative Commons Attribution License (https://creativecommons.org/licenses/by/4.0/).

4) The structural prediction of the CEP215 and Pericentrin interaction domains looks remarkably similar to the PReM-CM2 interaction for fly Cnn reported in Feng et al., 2017, Cell. Can the authors comment on this? Is this region of CEP215 the PReM domain? Does it also interact with the CM2 domain of CEP215? Could Pericentrin and CM2 be competing for binding to this region?

A number of previous reports indicate that CEP215 form homomultimers. In this manuscript, we specifically focused on the LZ:CM2 homotypic interaction which had been confirmed in *Drosophila* Cnn (Feng et al., 2017). Here, we performed coimmunoprecipitation assays with CEP215$^{611\text{-}670}$ and CEP215$^{1661\text{-}1893}$ which potentially correspond to the LZ and CM2 domains of human CEP215, respectively (Feng et al., 2017). First, we observed that both CEP215$^{611\text{-}670}$ and CEP215$^{1661\text{-}1893}$ formed homo-multimers, which may contribute homotypic interactions of CEP215 (Fig. 5E). However, HA-CEP215$^{611\text{-}670}$ was not coimmunoprecipitated with FLAG- CEP215$^{1661\text{-}1893}$, nor was HA-CEP215$^{1661\text{-}1893}$ with FLAG-CEP215$^{611\text{-}670}$ (Fig. 5E). These results suggest that the human CEP215 may not form the LZ:CM2 tetramer assembly which have been proposed in *Drosophila* Cnn (Feng et al., 2017). Rather, the CM2 domain which corresponds to CEP215$^{1661\text{-}1893}$ specifically interacts with PCNT in human (Figs 4 and 5).

5) When the authors use "phase separation mutations" to conclude that phase separation of CEP215 is important for centrosome and mitotic spindle assembly, they need to consider the possibility that they are mutating regions of Cep215 that interact with other PCM proteins - this means the failure to properly assemble centrosomes does not necessarily have anything to do with phase separation, as one cannot distinguish whether it is the ability of Cep215 to phase separate, or the ability of it to form important connections with other major PCM components.

Thank you for the critical comment. We agree that the CCD-truncation mutations might lead to failure of specific interactions of CEP215 with other PCM proteins. To test this possibility, we performed coimmunoprecipitation assays. We immunoprecipitated selected CCD mutants of CEP215 with FLAG antibodies, and detected coimmunoprecipitated endogenous PCM proteins, such as CEP215, CEP192 and PCNT. As expected, endogenous CEP215 protein was coimmunoprecipitated with the ectopic FLAG-CEP215 proteins (Fig. S7). Endogenous PCNT was also coimmunoprecipitated with ectopic CEP215 except CEP215^{L1867S} (Fig. S7). Finally, Endogenous CEP192 was coimmunoprecipitated with ectopic CEP215 except CEP215$^{\square BS1}$- CRY2, which lacks a portion of the CEP192-interacting domain (Kuriyama and Fisher, 2020; Fig. S7). The coimmunoprecipitation assays revealed that selected sequence-specific interactions are not affected in the CCD-truncated mutants of CEP215. Nonetheless, spindle pole assembly and functions are affected in the cells whose CEP215 is substituted with these CCD-truncated mutants (Figs. 8, 9). These results support the notion that molecular dynamics of CEP215 in spindle poles may be domain-specific rather than sequence-specific. The CCDs may be involved in multimeric interactions with other PCM proteins to assemble spindle poles and to maintain mitotic spindles. This point is described in the Discussion section of the revised manuscript.

Minor concerns

6) "Phase separation mechanisms have been implicated in assembly of centrosomes, non-membranous organelles (Jiang et al., 2021; Yeh et al., 2024)" - I think the Woodruff papers should be cited here as they were the first to show phase separation of a centrosome protein.

Thank you for the suggestion. We agree that the Woodruff papers should be more suitable.

7) The authors say that "the expression levels of ectopic FLAG-mCh-CEP215 were comparable to those of endogenous CEP215 in control cells (Fig. 1A)", but the ectopic CEP215 appears to be more strongly expressed - have the authors quantified this?

Quantification of the CEP215-specific bands in Figure 1A revealed that the amount of FLAG- mCh-CEP215 is approximately twice of endogenous CEP215 (Figure 1A). In addition, CEP215-specific signals at the mitotic centrosomes is about the same between endogenous CEP215 and FLAG-mCh-CEP215 (Figure 8). These data are included in the Figure 8 of the revised manuscript.

8) The authors say that "Similar to endogenous CEP215, FLAG-mCh-CEP215 was localized to the centrosomes, and the level of accumulation at the centrosome changed during the cell cycle, such that it was minimal at S phase, started to increase at G2 phase, and reached the highest level at M phase (Fig. 1B)." It seems to me that the highest level in both cases is in G2, not M phase. Have the authors quantified this?

© 2025. Published by The Company of Biologists under the terms of the Creative Commons Attribution License (https://creativecommons.org/licenses/by/4.0/).

We agree that the size of centrosomes at G2 phase looks larger than that at M phase. In fact, we previously reported that the centrosome sizes are larger in late G2 phase than those in mitosis (Lee and Rhee, 2010). We interpret the results that selective PCM proteins are recruited to the centrosomes at G2 phase, and reorganized at M phase for functional spindle pole assembly. We propose that CEP215 dynamics may be enhanced for the reorganization process of spindle poles. To test the hypothesis, we determined the sizes of centrosomes in HeLa cells with CEP215 mutant proteins during the cell cycle. The results showed that the centrosome areas of CEP215 and PCNT was enlarged at G2 phase and eventually reduced in M phase of the control HeLa cells, whereas those of γ-tubulin and CEP192 steadily increased as reported previously (Figs. 8D and S8). However, the centrosome sizes of the FLAG-mCh-CEP215$^{\square CCD5}$- and FLAG-mCh-CEP215^{L1867S}-expressing cells were generally reduced in the G2 phase and M phase cells (Fig. 8D). These results suggest that upregulation of the CEP215 dynamics is important for centrosome recruitment of PCM proteins during entering mitosis.

9) "Consistent with these, we found that cytoplasmic condensates of FLAG-CEP215F75A- CRY2 and FLAG-CEP215ΔCM2-CRY2 are devoid of γ-tubulin and PCNT signals, respectively (Fig. 4B, C)." From the picture, it seems as though g-tub is localising to the puncta in F75A mutants.
First of all, we changed to better representing images of Fig. 3B, so that one can clearly distinguish specific signals of FLAG, γ-tubulin and PCNT. As pointed out, co-localization of γ-tubulin and PCNT are diminished in cytoplasmic condensates of CEP215-CRY2^{F75A} and CEP215-CRY2$^{\Delta CM2}$, respectively (Fig. 3B). It is interesting that γ-tubulin is detected at the centrosomes in CEP215-CRY2^{F75A}-substituted cells, probably through other centrosome proteins, such as CEP192. We also observed microtubule re-grew from the condensates (Fig. S3).

10) "Interestingly, as we examined the light-induced condensation of CEP215 variants, we noticed that cytoplasmic condensation was highly reduced in the construct of CM2 deletion, FLAG-CEP215ΔCM2-CRY2, suggesting that CM2 is critical for the phase separation of CEP215 in cells (Fig. 4D; Fig. S2A). This result also implies that the association of PCNT, rather than the CM2-mediated self-association per se, may play an important role in the phase separation of CEP215." I'm not sure why this result would suggest a role for Pericentrin and not CM2 oligomerisation?
It may be hard to emphasize the importance of CEP215-PCNT interaction only with the CM2 deletion experiments. However, we have additional data which support importance of CEP215-PCNT interaction for phase separation. For example, we observed that cytoplasmic condensate formation was reduced in *PCNT*-deleted HeLa cells. We also observed similar phenotypes with CEP215 point mutants (CEP215^{L1860S} and CEP215^{L1867S}) which lack specific interaction with PCNT. The fact that two different point mutants revealed reduction in cytoplasmic condensation rates strongly suggest that PCNT interaction enhances CEP215 dynamics in mitotic centrosomes (Figure 5D). In the revised manuscript, we added experimental evidence which support that the CM2 domain of CEP215 is not involved in homotypic interactions (Fig. 5E). Therefore, we conclude that PCNT interaction is important for CEP215 phase separation.

11) "When the binding domains of CEP215 (MBP-CEP215 1814-1877) and PCNT (MBP- PCNT 2388-2420) were bacterially expressed, purified and mixed in vitro, they instantly form a complex (Fig. S3A)." These protein fragments are similar in size and so could be expected to elute from the SEC column at a similar volume even if not in a complex.
In the revised manuscript, we present an immunoblot analysis in both MBP-CEP215$^{1814-1877}$ and MBP-PCNT$^{2388-2420}$ were cofractionated in the size exclusion column (Fig. 4A). We also present SEC-MALS data revealing the molecular weight of the complex (Figure 4D).

12) "These results suggest that the intrinsic phase separation ability of CEP215 relies on intact CCD structures". So far, the authors have not shown that phase separation ability is intrinsic, as they have performed the experiments with a CRY2 domain.
FRAP recovery of mCh-CEP215 at the centrosome strongly support that molecular dynamics of CEP215 is upregulated at M phase. However, we did not perform in vitro experiments with purified proteins, due to the technical limitation. It is hard to observe liquid-like properties of CEP215 in vivo, since The CEP215 protein is concentrated into the centrosome. Therefore, we agree that we cannot definitely conclude that CEP215 undergo phase separation at M phase centrosomes. As suggested, we tone down our conclusion in the Discussion section of the revised

© 2025. Published by The Company of Biologists under the terms of the Creative Commons Attribution License (https://creativecommons.org/licenses/by/4.0/).

manuscript.

Second decision letter

MS ID#: jcs.263542R1

MS TITLE: Enhancement of CEP215/CDK5RAP2 dynamics for spindle pole assembly during mitosis

AUTHORS: Hyunjeong Yoo; Taehyun Kim; Sungjin Ryu; Donghee Ko; Jeesoo Kim; Hee-Jung Choi; Yongdae Shin; Kunsoo Rhee

ARTICLE TYPE: Research Article

Dear Dr Rhee,

We have now reached a decision on the above manuscript. Two of the previous reviewers have revised your manuscript. They agreed that the paper has improved but still considered that the claims made on phase separation are not adequate.

The reviewers raise a number of substantial criticisms that still require revision. I would advice that a revised version might prove acceptable, if you can address their concerns. Maybe consider taking into consideration the nomenclature used in the results section to describe very carefully the behaviour of CEP215 and then, if you wish considering using the possibility of discussing this behavior in the discussion, as suggested by one of the reviewers. If you think that you can deal satisfactorily with the criticisms on revision, I would be pleased to see a revised manuscript. We would then return it to the reviewers.

Reviewer 1

Advance summary and potential significance to field

This paper characterizes the importance of various domains and heterotypic interactions in CEP215 that are important for PCM assembly in human cells. This work helps understand how the PCM scaffold assembles and achieves maximum size in preparation for mitosis. The authors still claim that CEP215 assembles through phase separation, but this claim is not fully supported and is derived from an artificial light-induced multimerization system.

Comments for the author

The authors have addressed most of my criticisms, but a few key criticisms remain. The authors somewhat downplay the conclusion that Cep215 can self-assemble through phase separation, yet the claim still shows up in various parts of the paper. In the revised version, the authors focus on the dynamic properties of Cep215 during the cell cycle and how they are influenced by mutations within multimerization domains and protein-binding domains. While I find acceptable the experiments and analyses of the various Cep215 mutants, I do not agree with the authors' interpretations. For example, in the abstract they state:

"Our results support the notion that the diffusible mobility of CEP215 is enhanced by both homotypic and heterotypic interactions among CCDs, especially at mitotic spindle poles."

There is no strong evidence that an increase in homotypic and heterotypic interactions leads to a faster mobility (i.e., higher diffusion coefficient) with the PCM. Rather, the authors use FRAP to show that mCherry-tagged CEP215 mutants, most of which form small PCM, do not exchange between the cytoplasm and the PCM. At first glance, this seems like a reasonable interpretation. However, the authors show in Figure 1 that CEP215 is not dynamic in the interphase PCM, which is

© 2025. Published by The Company of Biologists under the terms of the Creative Commons Attribution License (https://creativecommons.org/licenses/by/4.0/).

smaller than mitotic PCM. It is possible that the various CEP215 mutations prevent formation of mitotic PCM. If true, one would instead conclude that multiple coiled-coil domains and PCNT-binding sites within Cep215 are required for PCM expansion. Thus, the main conclusion that functional spindle pole assembly relies on enhanced CEP215 dynamics is not adequately supported by the data.

My main assessment of the paper, like before, is that this paper is worthwhile and should be published. However, the authors need to revise their interpretations and consider alternative explanations.

I have additional concerns and comments:

1. Main text. "We found that CEP215 has an intrinsic phase-separation property."

This statement is not supported by the data. The authors never show that full-length CEP215 can phase separate on its own without the Cry2 system.

2. Figure 1E. How were the solid lines created? What do they represent? Are the authors fitting the data? This is not mentioned in the legend. To me, the lines look hand drawn.

3. Figure 2A. I can barely see the opto-droplets. Part of the problem is the red coloration. My eyes can't see that color of red well. Perhaps the authors could make the images brighter or switch to magenta or red-orange.

4. Figure 2A. Are the mCh-CEP215 assemblies dynamic (as assessed by FRAP) or do they coalesce? If they do, this suggests that the Cry2 tag is not required for phase separation of CEP215. However, if these assemblies are not dynamic and don't coalesce, then it means that the Cry2 is responsible for the liquid-like behavior. We have seen that GFP and other well-behaved monomeric tags can influence the phase behavior of proteins, so this is a legitimate concern. The fact that Cry2 on its own does not form dynamic condensates is not sufficient to address this concern (see: PMID: 38141760, PMID: 38141760). This is why I think the CRY2 system is not a legitimate way to test if a protein undergoes phase separation.

5. Main text. "Even if CEP215 is a centrosome protein, we found that excess amounts of the ectopic mCh-CEP215-CRY2 proteins were transiently accumulated at the cytoplasm."

I don't understand this sentence. Are the authors saying that mCh-CEP215-CRY2 formed assemblies in the cytoplasm? Or are they saying the mCH-CEP215 signal became visible in the cytoplasm after expression was induced? The authors did not reference a figure panel for this statement.

6. Main Text. "When the binding domains of CEP215 (MBP-CEP2151814-1877) and PCNT (MBP-PCNT2388-2420) were bacterially expressed, purified and mixed in vitro, they instantly form a complex (Fig. 4A)."

Overlapping elution profiles in SEC is not evidence of complex formation. I would be more convinced if the authors showed the elution profiles of each protein individually, so that I could compare. However, this is not necessary, as the ITC experiments are definitive proof of complex formation. Thus, I suggest rewording this sentence to be more accurate.

7. "Finally, SEC-MALS analysis of MBP-CEP2151661-1893 and PCNT2350-2450 revealed that the CEP215 and PCNT fragments form 2:2 tetramer (Fig. 4D)"

The writing here needs significant improvement. This sentence does not describe what was done in figure 4D. Figure 4D demonstrates that MBP-CEP2151661-1893 and PCNT2350-2450 each form dimers. Then, based on the ITC data, one reasons that these fragments come together to form a tetramer.
Related to this point, the description of Figure 4 seems backward to me. It would be more logical to describe the assembly properties of each protein individually, then demonstrate that they form a complex. I would rearrange it this way: Panel D first, followed by A, B, then C.

© 2025. Published by The Company of Biologists under the terms of the Creative Commons Attribution License (https://creativecommons.org/licenses/by/4.0/).

8. Main text. "A number of previous reports indicate that CEP215 forms homomultimers." I'm not aware of this. Please cite the papers that specifically reference that human Cep215 multimerizes. The only data I can recall are demonstrations that the CM1 domain can form dimers. The most in-depth analyses of PCM scaffold multimerization come from Cnn and SPD-5.

9. Figure 6E. I find these experiments interesting, but I'm worried that the authors have misinterpreted the data. They claim that the Cep215 mutations reduce PCM dynamicity, and that gives rise to the cellular phenotypes. An alternative explanation is that the mutations reduce Cep215 assembly, which is evidenced by smaller PCM shown in the figure. In this case, the interphase fraction of Cep215 still assembles, but mitotic fraction of Cep215 does not. Thus, in L1867S or ï¤„CCD5 mutants, for example, we could be looking at centrosomes with interphase-structured PCM. The authors showed in Figure 1 that interphase Cep215 is non-dynamic, which supports my concern. Can the authors point out cases where Cep215 dynamics are perturbed but assembly is not? Perhaps the CCD2 mutant fits this? If true, this would support the authors claim.

Without addressing my concern, the following statements are not justified:
"These results further suggest that CEP215 should dynamically interact with PCM proteins for the proper formation of mitotic spindles."

"We propose that diffusive mobility of CEP215 is controlled by both homotypic and heterotypic interactions among CCDs, especially at mitotic spindle poles."

"We found that selected CCD-truncated mutants failed to rescue spindle defects in the CEP215 KO cells, suggesting that upregulation of CEP215 mobility is essential for proper spindle pole functions (Fig. 7E)."

"Our results support the notion that the diffusible mobility of CEP215 is enhanced by both homotypic and heterotypic interactions among CCDs, especially at mitotic spindle poles."

10. "We found that CEP215 has an intrinsic phase-separation capability in help of the multimerizing CRY2 domain."

This sentence is not accurate, and maybe it's the phrasing that is off. There is no demonstration in this paper that Cep215 has an intrinsic ability to phase separate. To me, "intrinsic" means "by itself", without the help of non-natural fusion tags. I would state: "We found that, when fused to a multimerizing CRY2 domain, CEP215 forms cytoplasmic, liquid-like assemblies".

11. Figure S7. Some panels are missing the bleaching ROI. Also, the legend mentions a statistical test, but I don't see any asterisks in the plot.

<u>Reviewer 2</u>

Advance summary and potential significance to field

The paper is improved but there remain missing controls and overstatements and conclusions that are not supported by the data. The paper cannot be published until the issues below are taken into account and in the end I feel the interest of the findings will not be sufficient to be published in a broad-scope journal such as JCS.

Major comments

1) The first version of the paper made bold claims that were not supported by the data, centring around the claim that CEP215 has an intrinsic ability to phase separate. The Reviewers took issue with this as the authors had used a light inducible CRY2 dimerisation domain to force the clustering of CEP215 aggregates in cells, which does not show CEP215 can phase separate nor does it show CEP215 has an intrinsic ability to phase separate. They were therefore asked to tone down their conclusions or show that CEP215 really does have an intrinsic propensity to phase separate. The authors point out in their response to reviewers that they have been unable to "purify biologically active CEP215 proteins" and so could not test the intrinsic property for CEP215 to phase separate. They have changed the title and toned down the abstract to avoid referring to an

© 2025. Published by The Company of Biologists under the terms of the Creative Commons Attribution License (https://creativecommons.org/licenses/by/4.0/).

intrinsic propensity to phase-separate, but they regularly use these terms throughout the rest of the text. E.g. "We found that CEP215 has an intrinsic phase-separation property…To probe the intrinsic phase-separation capacity of CEP215, we employed the opto- droplet system (Shin et al., 2017).These results suggest that the intrinsic phase separation ability of CEP215 relies on intact CCD structures… We found that CEP215 has an intrinsic phase-separation capability in help of the multimerizing CRY2 domain… To further confirm the importance of PCNT in CEP215 phase separation…" This is all very misleading and the wording should be altered throughout. It would be better to say something like "the light-inducible clustering propensity of CEP215 was affected when XXX domain was mutated". Then, only in the discussion can the authors suggest that CEP215 may phase separate in cells and that their data would fit with the idea that the domains they have identified are involved in such a putative phase separation.

2) With regard to how the authors say that the light-induced mCh-CEP215-CRY2 clusters showed characteristics of liquid-like condensates as they often grew in size through fusion and showed shape relaxation behaviors after fusion (Fig. 2B), from the movie, it seems that often 2 blobs will overlap but then separate, which would suggest they are not liquid-like…can this be quantified? What proportion of interaction events result in fusion?

3) Sometimes the authors referring to the light-induced clusters as condensates and sometimes as clusters. They need to be consistent and given that they cannot be sure they are phase-separated condensates, it would be safer to refer to them as clusters. This includes when saying "These results suggest that PCNT interaction at the CM2 domain of CEP215 is critical for its cytoplasmic condensate formation" - they can say it is important for light-induced clustering.

4) At the end of the second results section, the authors state: "Taken together, the opto-droplet assay demonstrated that CEP215 has a capacity to undergo phase separation in cells." I think this is a massive overstatement. They don't know this is phase separation as opposed to simple clustering. And the clustering is being induced by the Cry2 domain!!

5) The authors say: "Consistent with these, we found that cytoplasmic condensates of FLAG-CEP215F75A-CRY2 and FLAG-CEP215Î”CM2-CRY2 are devoid of Î³-tubulin and PCNT signals, respectively (Fig. 3B, C)." This is simply not true. The quantification shows that over 50% of cells have clusters that associate with either Î³-tubulin or PCNT.

6) When examining the in vitro phase separation properties of the mixture of MBP-CEP2151814-1877 and MBP- PCNT2388-2420, the authors need to perform the control where they show that these fragments do not phase separate alone. This is especially important for CEP2151814-1877, as the authors claim that the interaction with PCNT2388-2420 is important for CEP215 phase separation. The authors should also test whether fragments containing the interaction mutations phase separate or not. These controls will help address whether any old protein fragment will phase separate in vitro under crowding conditions.

7) Fig 4D: The authors claim that SEC-MALS shows that the fragments show a 2:2 tetramer BUT THIS IS NOT WHAT IS SHOWN! They show that each individual fragment forms a dimer. Why have they not done SEC-MALS on the mixed fragment sample?

8) The authors say that "Coimmunostaining analyses also revealed that PCNT is hardly detected at the cytoplasmic condensates of FLAG-CEP215L1860S-CRY2 and FLAG-CEP215L1867S-CRY2 (Fig. 5C)" - but in the figure it looks to be clearly recruited.

9) The authors main claim is that the dynamicity of CEP215 is critical for centrosome accumulation of PCM, but can the authors really relate the defects they see to reduced dynamics of CEP215? When they mutate CEP215, they are not necessarily affecting only dynamics of CDK5RAP2 - they are interfering with the interaction with PCNT and possibly with other PCM proteins that they have not analysed. I feel like this conclusion does not have a solid enough base from the current data.

10) The authors state that they "clearly demonstrated that the interaction with PCNT is crucial for diffusive mobility of CEP215" but how do the authors imagine the interaction can increase the diffusive mobility of CEP215? It seems like an interaction would slow down rather than increase dynamics...?

Minor comments

1) Any red/green image overlay eg. fig S1 should be changed for colour-blind friendly colours.

2) The authors should show some example images for Figure 3E.

3) "Immunoblot analysis revealed that all the CEP215 mutants were expressed at comparable levels to the endogenous CEP215 (Fig. 6D)". This is difficult to assess as all the bands are saturated. The authors need to provide an image where the bands are not saturated.

© 2025. Published by The Company of Biologists under the terms of the Creative Commons Attribution License (https://creativecommons.org/licenses/by/4.0/).

Second **revision**

Author response to reviewers' comments

Reviewer 1: SUMMARY OF THE ADVANCE MADE IN THIS PAPER AND ITS POTENTIAL SIGNIFICANCE TO THE FIELD

This paper characterizes the importance of various domains and heterotypic interactions in CEP215 that are important for PCM assembly in human cells. This work helps understand how the PCM scaffold assembles and achieves maximum size in preparation for mitosis. The authors still claim that CEP215 assembles through phase separation, but this claim is not fully supported and is derived from an artificial light-induced multimerization system.

SUGGESTIONS TO AUTHORS

The authors have addressed most of my criticisms, but a few key criticisms remain. The authors somewhat downplay the conclusion that Cep215 can self-assemble through phase separation, yet the claim still shows up in various parts of the paper. In the revised version, the authors focus on the dynamic properties of Cep215 during the cell cycle and how they are influenced by mutations within multimerization domains and protein-binding domains. While I find acceptable the experiments and analyses of the various Cep215 mutants, I do not agree with the authors' interpretations. For example, in the abstract they state:
"Our results support the notion that the diffusible mobility of CEP215 is enhanced by both homotypic and heterotypic interactions among CCDs, especially at mitotic spindle poles."

There is no strong evidence that an increase in homotypic and heterotypic interactions leads to a faster mobility (i.e., higher diffusion coefficient) with the PCM. Rather, the authors use FRAP to show that mCherry-tagged CEP215 mutants, most of which form small PCM, do not exchange between the cytoplasm and the PCM. At first glance, this seems like a reasonable interpretation. However, the authors show in Figure 1 that CEP215 is not dynamic in the interphase PCM, which is smaller than mitotic PCM. It is possible that the various CEP215 mutations prevent formation of mitotic PCM. If true, one would instead conclude that multiple coiled-coil domains and PCNT-binding sites within Cep215 are required for PCM expansion. Thus, the main conclusion that functional spindle pole assembly relies on enhanced CEP215 dynamics is not adequately supported by the data.

My main assessment of the paper, like before, is that this paper is worthwhile and should be published. However, the authors need to revise their interpretations and consider alternative explanations.
We are grateful for the reviewer’s insightful feedback and constructive suggestions. We accept that our manuscript primarily reports the clustering properties of CEP215 in vivo, rather than definitively demonstrating phase separation. In response to the reviewer’s comments, we have carefully revised the manuscript, ensuring that we focus on the clustering dynamics of CEP215 and provide a more accurate interpretation of the data. Additionally, we have revised the results section to include alternative explanations for the centrosome FRAP data, as suggested by the reviewer. We now emphasize that the reduced mobility observed in some CEP215 mutants may be due to impaired PCM formation rather than changes in dynamic behavior. These revisions should clarify our conclusions and better align with the data presented.

I have additional concerns and comments:

1. Main text. "We found that CEP215 has an intrinsic phase-separation property."

This statement is not supported by the data. The authors never show that full-length CEP215 can phase separate on its own without the Cry2 system.

© 2025. Published by The Company of Biologists under the terms of the Creative Commons Attribution License (https://creativecommons.org/licenses/by/4.0/).

We accept that Fig. 2 does not directly demonstrate an intrinsic property of CEP215. Rather, it shows that ectopic CEP215 exhibits liquid-like properties when linked to the CRY2 domain. In light of this, we have revised the manuscript to clarify this point: “Live-imaging revealed that mCh-CEP215-CRY2 instantly formed clusters in the cytoplasm upon light activation (Fig. 2A; Movie 1).

2. Figure 1E. How were the solid lines created? What do they represent? Are the authors fitting the data? This is not mentioned in the legend. To me, the lines look hand drawn.
We apologize for the lack of clarity regarding Fig. 1E. The solid lines represent regression curves generated using the Prizm software. We have revised the figure legend in the manuscript to clearly explain this.

3. Figure 2A. I can barely see the opto-droplets. Part of the problem is the red coloration. My eyes can't see that color of red well. Perhaps the authors could make the images brighter or switch to magenta or red-orange.
As suggested, we have changed the color to bright yellow to improve visibility. Additionally, Fig. 2A is also shown in Movie 1 for further reference.

4. Figure 2A. Are the mCh-CEP215 assemblies dynamic (as assessed by FRAP) or do they coalesce? If they do, this suggests that the Cry2 tag is not required for phase separation of CEP215. However, if these assemblies are not dynamic and don't coalesce, then it means that the Cry2 is responsible for the liquid-like behavior. We have seen that GFP and other well- behaved monomeric tags can influence the phase behavior of proteins, so this is a legitimate concern. The fact that Cry2 on its own does not form dynamic condensates is not sufficient to address this concern (see: PMID: 38141760). This is why I think the CRY2 system is not a legitimate way to test if a protein undergoes phase separation.
Thank you for your thoughtful comment. We found that most of the mCh-CEP215 aggregates are neither dynamic nor coalesce, whereas mCh-CEP215-CRY2 clusters form and coalesce upon light activation. These results suggest that the CRY2 domain facilitates the concentration of mCh-CEP215, promoting a liquid-like state. Additionally, we observed that mCh-CEP215- CRY2 clusters form at moderate expression levels, while mCh-CEP215 aggregates are largely absent (Fig. S2). While these findings suggest that CRY2 may play a key role in promoting dynamic clustering, we cannot rule out the possibility that the CRY2 domain itself influences the formation of dynamic clusters. Therefore, we have carefully interpreted the data from Fig. 2A in this context.

5. Main text. "Even if CEP215 is a centrosome protein, we found that excess amounts of the ectopic mCh-CEP215-CRY2 proteins were transiently accumulated at the cytoplasm."
I don't understand this sentence. Are the authors saying that mCh-CEP215-CRY2 formed assemblies in the cytoplasm? Or are they saying the mCH-CEP215 signal became visible in the cytoplasm after expression was induced? The authors did not reference a figure panel for this statement.
To clarify, we meant that “mCh-CEP215-CRY2 signals became visible in the cytoplasm, in addition to the centrosomes (Fig. 2A). Live-imaging revealed that mCh-CEP215-CRY2 instantly formed clusters in the cytoplasm upon light activation (Fig. 2A; Movie 1).”

6. Main Text. "When the binding domains of CEP215 (MBP-CEP215$^{1814\text{-}1877}$) and PCNT (MBP-PCNT$^{2388\text{-}2420}$) were bacterially expressed, purified and mixed in vitro, they instantly form a complex (Fig. 4A)."
Overlapping elution profiles in SEC is not evidence of complex formation. I would be more convinced if the authors showed the elution profiles of each protein individually, so that I could compare. However, this is not necessary, as the ITC experiments are definitive proof of complex formation. Thus, I suggest rewording this sentence to be more accurate.
We appreciate for your suggestion for a more accurate description of the results. As recommended, we have rewritten the sentence as follows. “When MBP-CEP215$^{1814\text{-}1877}$ and MBP-PCNT$^{2388\text{-}2420}$ were mixed in solution and subjected to the SEC analysis, both the proteins were eluted at the same fractions, suggesting that they instantly form a heterogenous complex in vitro (Fig. 4B).”

© 2025. Published by The Company of Biologists under the terms of the Creative Commons Attribution License (https://creativecommons.org/licenses/by/4.0/).

7. "Finally, SEC-MALS analysis of MBP-CEP215$^{1661-1893}$ and PCNT$^{2350-2450}$ revealed that the CEP215 and PCNT fragments form 2:2 tetramer (Fig. 4D)"
The writing here needs significant improvement. This sentence does not describe what was done in figure 4D. Figure 4D demonstrates that MBP-CEP215$^{1661-1893}$ and PCNT$^{2350-2450}$ each form dimers. Then, based on the ITC data, one reasons that these fragments come together to form a tetramer.
Related to this point, the description of Figure 4 seems backward to me. It would be more logical to describe the assembly properties of each protein individually, then demonstrate that they form a complex. I would rearrange it this way: Panel D first, followed by A, B, then C.
We appreciate your suggestion. As recommended, we have reorganized Fig. 4, and rewritten the sentence for clarity. We believe this revised description now presents the data in a more logical and coherent manner.

8. Main text. "A number of previous reports indicate that CEP215 forms homomultimers."
I'm not aware of this. Please cite the papers that specifically reference that human Cep215 multimerizes. The only data I can recall are demonstrations that the CM1 domain can form dimers. The most in-depth analyses of PCM scaffold multimerization come from Cnn and SPD- 5.
We acknowledge that the evidence for homo-multimerization of CEP215-like proteins comes from the study by Feng et al. (2017). In light of this, we revised as follow: “It was previously reported that centrosomin (Cnn), a *Drosophila* homologue of CEP215, forms homo-multimers (Feng et al., 2017).”

9. Figure 6E. I find these experiments interesting, but I'm worried that the authors have misinterpreted the data. They claim that the Cep215 mutations reduce PCM dynamicity, and that gives rise to the cellular phenotypes. An alternative explanation is that the mutations reduce Cep215 assembly, which is evidenced by smaller PCM shown in the figure. In this case, the interphase fraction of Cep215 still assembles, but mitotic fraction of Cep215 does not. Thus, in L1867S or CCD5 mutants, for example, we could be looking at centrosomes with interphase-structured PCM. The authors showed in Figure 1 that interphase Cep215 is non- dynamic, which supports my concern. Can the authors point out cases where Cep215 dynamics are perturbed but assembly is not? Perhaps the CCD2 mutant fits this? If true, this would support the authors claim.
Without addressing my concern, the following statements are not justified:
"These results further suggest that CEP215 should dynamically interact with PCM proteins for the proper formation of mitotic spindles."
"We propose that diffusive mobility of CEP215 is controlled by both homotypic and heterotypic interactions among CCDs, especially at mitotic spindle poles."
"We found that selected CCD-truncated mutants failed to rescue spindle defects in the CEP215 KO cells, suggesting that upregulation of CEP215 mobility is essential for proper spindle pole functions (Fig. 7E)."
"Our results support the notion that the diffusible mobility of CEP215 is enhanced by both homotypic and heterotypic interactions among CCDs, especially at mitotic spindle poles."
We agree that it is possible that the reduction in mitotic PCM size may, at least in part, contribute to the failure to recover fluorescent signals from the CEP215 mutant proteins. In the revised manuscript, we have added an explanation of this possibility as follows: “Another possibility is that the truncated CEP215 mutant proteins are not efficiently recruited to mitotic centrosomes (See Fig. 7). In this case, the reduced FRAP activity may partly result from the low molecular dynamics of mCh-CEP215, which persists from interphase centrosomes.” This revised wording reflects our consideration of the alternative explanation and makes it clear that both dynamic properties and PCM assembly are important for the proper functioning of CEP215 at the centrosome. Thank you for your thoughtful feedback.

10. "We found that CEP215 has an intrinsic phase-separation capability in help of the multimerizing CRY2 domain."
This sentence is not accurate, and maybe it's the phrasing that is off. There is no demonstration in this paper that Cep215 has an intrinsic ability to phase separate. To me, "intrinsic" means "by itself", without the help of non-natural fusion tags. I would state: "We found that, when fused to a multimerizing CRY2 domain, CEP215 forms cytoplasmic, liquid-like assemblies".
We appreciate for the suggestion. We rewrote the text as follow: “Taken together, the opto-droplet assay demonstrated that CEP215 undergoes light-induced clustering when linked to the CRY2 domain”.

© 2025. Published by The Company of Biologists under the terms of the Creative Commons Attribution License (https://creativecommons.org/licenses/by/4.0/).

11. Figure S7. Some panels are missing the bleaching ROI. Also, the legend mentions a statistical test, but I don't see any asterisks in the plot.
We are shamed of our sloppiness. We added ROI is marked at every image of Fig. S6. We did not perform statistical tests in the FRAP analysis.

Reviewer 3: The paper is improved but there remain missing controls and overstatements and conclusions that are not supported by the data. The paper cannot be published until the issues below are taken into account and in the end I feel the interest of the findings will not be sufficient to be published in a broad-scope journal such as JCS.
Major comments
1) The first version of the paper made bold claims that were not supported by the data, centering around the claim that CEP215 has an intrinsic ability to phase separate. The Reviewers took issue with this as the authors had used a light inducible CRY2 dimerisation domain to force the clustering of CEP215 aggregates in cells, which does not show CEP215 can phase separate nor does it show CEP215 has an intrinsic ability to phase separate. They were therefore asked to tone down their conclusions or show that CEP215 really does have an intrinsic propensity to phase separate. The authors point out in their response to reviewers that they have been unable to "purify biologically active CEP215 proteins" and so could not test the intrinsic property for CEP215 to phase separate. They have changed the title and toned down the abstract to avoid referring to an intrinsic propensity to phase-separate, but they regularly use these terms throughout the rest of the text. E.g. "We found that CEP215 has an intrinsic phase-separation property…To probe the intrinsic phase-separation capacity of CEP215, we employed the opto- droplet system (Shin et al., 2017). These results suggest that the intrinsic phase separation ability of CEP215 relies on intact CCD structures…We found that CEP215 has an intrinsic phase-separation capability in help of the multimerizing CRY2 domain… To further confirm the importance of PCNT in CEP215 phase separation…" This is all very misleading and the wording should be altered throughout. It would be better to say something like "the light- inducible clustering propensity of CEP215 was affected when XXX domain was mutated". Then, only in the discussion can the authors suggest that CEP215 may phase separate in cells and that their data would fit with the idea that the domains they have identified are involved in such a putative phase separation.
We appreciate the reviewer's careful evaluation of our manuscript. As noted, our study examines the clustering properties of CEP215 in vivo rather than demonstrating its intrinsic ability to phase separate. In response to the reviewer's feedback, we have thoroughly revised the manuscript to ensure that our conclusions are accurately represented. Following the reviewer's suggestions, we have removed language that implies an intrinsic phase-separation ability of CEP215 throughout the manuscript. Instead, we now describe our findings in a neutral manner, focusing on the observed clustering behavior. Interpretations regarding the potential role of CEP215 in phase separation are now explicitly reserved for the discussion section, where we carefully frame them as a hypothesis rather than a definitive conclusion.

2) With regard to how the authors say that the light-induced mCh-CEP215-CRY2 clusters showed characteristics of liquid-like condensates as they often grew in size through fusion and showed shape relaxation behaviors after fusion (Fig. 2B), from the movie, it seems that often 2 blobs will overlap but then separate, which would suggest they are not liquid-like…can this be quantified? What proportion of interaction events result in fusion?
The movie 1 shows that dispersed fluorescent signals rapidly form clusters within the first 30 seconds. Over time, we observe both fusion and separation events, but overall, clusters enlarge significantly by 300 seconds (Fig. 2B). We believe that such clustering and enlargement behaviors suggest a liquid-like character of the mCh-CEP215-CRY2 clusters in the cytoplasm.

3) Sometimes the authors referring to the light-induced clusters as condensates and sometimes as clusters. They need to be consistent and given that they cannot be sure they are phase-separated condensates, it would be safer to refer to them as clusters. This includes when saying "These results suggest that PCNT interaction at the CM2 domain of CEP215 is critical for its cytoplasmic condensate formation" - they can say it is important for light-induced clustering.
We appreciate the reviewer's feedback and understand the importance of precise terminology. As suggested, we have replaced the term 'condensates' with 'clusters' throughout the manuscript to ensure consistency and avoid implying phase separation where it has not been definitively demonstrated.

© 2025. Published by The Company of Biologists under the terms of the Creative Commons Attribution License (https://creativecommons.org/licenses/by/4.0/).

4) At the end of the second results section, the authors state: "Taken together, the opto-droplet assay demonstrated that CEP215 has a capacity to undergo phase separation in cells." I think this is a massive overstatement. They don't know this is phase separation as opposed to simple clustering. And the clustering is being induced by the Cry2 domain!!

As suggested, we have revised our conclusion to more accurately reflect our findings. The statement now reads: 'Taken together, the opto-droplet assay demonstrated that CEP215 undergoes light-induced clustering when linked to the CRY2 domain.' This revision ensures that we do not overstate our findings and more precisely describe the observed phenomenon.

5) The authors say: "Consistent with these, we found that cytoplasmic condensates of FLAG-CEP215^{F75A}-CRY2 and FLAG-CEP215$^{\Delta CM2}$-CRY2 are devoid of γ-tubulin and PCNT signals, respectively (Fig. 3B, C)." This is simply not true. The quantification shows that over 50% of cells have clusters that associate with either γ-tubulin or PCNT.

We acknowledge that our original statement overstated the lack of association of FLAG-CEP215^{F75A}-CRY2 and FLAG-CEP215$^{\Delta CM2}$-CRY2 clusters with γ-tubulin and PCNT, respectively. We carefully described Fig. 3C as follow: "Our analysis shows that about 60% of cells containing FLAG-CEP215-CRY2 clusters displayed both γ-tubulin and PCNT signals, whereas fewer than 20% of cells expressing FLAG-CEP215^{F75A}-CRY2 or FLAG- CEP215$^{\Delta CM2}$-CRY2 contained both signals in the clusters. (Fig. 3C). Majority of the FLAG- CEP215^{F75A}-CRY2 and FLAG-CEP215$^{\Delta CM2}$-CRY2 clusters selectively devoid of γ-tubulin and PCNT, respectively (Fig. 3C)." This revision ensures that the manuscript accurately reflects the quantification and avoids misrepresentation of the data.

6) When examining the in vitro phase separation properties of the mixture of MBP-CEP215$^{1814-1877}$ and MBP- PCNT$^{2388-2420}$, the authors need to perform the control where they show that these fragments do not phase separate alone. This is especially important for CEP215$^{1814-1877}$, as the authors claim that the interaction with PCNT$^{2388-2420}$ is important for CEP215 phase separation. The authors should also test whether fragments containing the interaction mutations phase separate or not. These controls will help address whether any old protein fragment will phase separate in vitro under crowding conditions.

As mentioned, we initially attempted to crystallize the MBP-CEP215$^{1814-1877}$ and MBP- PCNT$^{2388-2420}$ complex to resolve its 3D structure. However, the mixture did not form crystals, which suggests that the complex may adopt a flexible configuration. Based on these challenges, we agree that we cannot conclusively assert that the complex undergoes phase separation solely from the data presented in Fig. 4D. However, our biophysical analyses in Fig. 4 do suggest that the MBP-CEP215$^{1814-1877}$ and MBP- PCNT$^{2388-2420}$ complex forms a tetrameric structure, and AlphaFold prediction provide valuable insights into the molecular interactions within the complex. We have updated the manuscript to clarify these points and emphasize that while phase separation cannot be definitively concluded.

7) Fig 4D: The authors claim that SEC-MALS shows that the fragments show a 2:2 tetramer BUT THIS IS NOT WHAT IS SHOWN! They show that each individual fragment forms a dimer. Why have they not done SEC-MALS on the mixed fragment sample?

We agree that Fig. 4D indicates that MBP-CEP215 and MBP-PCNT form dimers in solution. We predicted tetramer since the MBP-CEP215$^{1814-1877}$ and MBP- PCNT$^{2388-2420}$ proteins are eluted at the same fractions as shown in Fig. 4B. In the revised manuscript, we arranged the data in logical orders and correctly described the facts that the reviewer pointed out.

8) The authors say that "Coimmunostaining analyses also revealed that PCNT is hardly detected at the cytoplasmic condensates of FLAG-CEP215^{L1860S}-CRY2 and FLAG- CEP215^{L1867S}-CRY2 (Fig. 5C)" - but in the figure it looks to be clearly recruited.

We appreciate the reviewer pointing out this inconsistency. To correct this, we replaced the FLAG-CEP215^{L1867S}-CRY2 group to a more representative sample. Additionally, we have marked the centrosomes with arrowheads in Fig. 5C to clarify that the PCNT signals overlap with γ-tubulin signals at the centrosomes. We hope that these changes improve the clarity of Fig. 5C and provide more accurate representation of the data.

9) The authors main claim is that the dynamicity of CEP215 is critical for centrosome accumulation of PCM, but can the authors really relate the defects they see to reduced dynamics of CEP215? When they mutate CEP215, they are not necessarily affecting only dynamics of CDK5RAP2 - they are interfering with the interaction with PCNT and possibly with other PCM

© 2025. Published by The Company of Biologists under the terms of the Creative Commons Attribution License (https://creativecommons.org/licenses/by/4.0/).

proteins that they have not analyzed. I feel like this conclusion does not have a solid enough base from the current data.
FRAP analysis revealed reduced dynamics of the mutant proteins both in the cytoplasm (Fig. 6C) and at the centrosome (Fig. 6E, F). Furthermore, mitotic spindle defects were observed in cells where endogenous CEP215 was substituted with the mutant forms (Fig. 8). Based on these observations, we propose that the reduced dynamics of CEP215 contribute to the mitotic spindle defects, possibly due to inefficient recruitment of CEP215 to the mitotic centrosomes (Fig. 7). We have revised the discussion section of the manuscript to clarify this point and emphasize that, while we cannot rule out other potential contributing factors, the data support the hypothesis that reduced CEP215 dynamics are critical for the observed defects

10) The authors state that they "clearly demonstrated that the interaction with PCNT is crucial for diffusive mobility of CEP215" but how do the authors imagine the interaction can increase the diffusive mobility of CEP215? It seems like an interaction would slow down rather than increase dynamics...?
We hypothesize that the CEP215-PCNT complex may maintain a flexible configuration, which could explain why it does not form crystals in vitro even at high concentrations (Fig. 4C). This flexibility may also be important for the dynamic behavior of the complex in the cellular context. We have added this point to the results section of the revised manuscript.

Minor comments
1) Any red/green image overlay eg. fig S1 should be changed for colour-blind friendly colours.
As suggested, we changed the overlay images in Fig. S1 are changed to magenta, cyan and yellow.

2) The authors should show some example images for Figure 3E. As suggested, we present example images of Fig. 5E in Fig. S2B.

3) "Immunoblot analysis revealed that all the CEP215 mutants were expressed at comparable levels to the endogenous CEP215 (Fig. 6D)". This is difficult to assess as all the bands are saturated. The authors need to provide an image where the bands are not saturated.
As suggested, the Fig. 6D blot image was changed with a less exposed one in the revised manuscript.

Third decision letter

MS ID#: jcs.263542R2

MS TITLE: Enhancement of CEP215/CDK5RAP2 dynamics for spindle pole assembly during mitosis

AUTHORS: Hyunjeong Yoo; Taehyun Kim; Sungjin Ryu; Donghee Ko; Jeesoo Kim; Hee-Jung Choi; Yongdae Shin; Kunsoo Rhee

ARTICLE TYPE: Research Article

Dear Dr Rhee,

We have now reached a decision on the above manuscript. I have read the rebuttal letter and the article and considered that you have addressed all reviewer comments, in particular by toning down the conclusions related with phase-separation.

This means that JCS will be happy to accept this publication after addressing the following points:

Please review the figures as many Figure panels lack scale bars.

Please provide new key words to replace phase separation .

© 2025. Published by The Company of Biologists under the terms of the Creative Commons Attribution License (https://creativecommons.org/licenses/by/4.0/).

I hope that you will be able to carry these out because I would like to be able to accept your paper, depending on further comments from reviewers.

Fourth decision letter

MS ID#: jcs.263542R3

MS Title: Enhancement of CEP215/CDK5RAP2 dynamics for spindle pole assembly during mitosis

Authors: Hyunjeong Yoo; Taehyun Kim; Sungjin Ryu; Donghee Ko; Jeesoo Kim; Hee-Jung Choi; Yongdae Shin; Kunsoo Rhee

Article Type: Research Article

Dear Dr Rhee,

I am happy to tell you that your manuscript has been accepted for publication in Journal of Cell Science, pending standard publication integrity checks. It was accepted on 15 Apr 2025. Where referee reports on this version are available, they are appended below.

© 2025. Published by The Company of Biologists under the terms of the Creative Commons Attribution License (https://creativecommons.org/licenses/by/4.0/).
